# Randomised controlled trials of antidepressant and anti-anxiety medications for people with autism spectrum disorder: systematic review and meta-analysis

**DOI:** 10.1192/bjo.2021.1003

**Published:** 2021-10-01

**Authors:** Shoumitro Deb, Meera Roy, Rachel Lee, Madiha Majid, Bharati Limbu, Jacopo Santambrogio, Ashok Roy, Marco O. Bertelli

**Affiliations:** Department of Brain Sciences, Faculty of Medicine, Imperial College London, UK; Department of Psychiatry, Hereford and Worcestershire Health and Care NHS Trust, UK; Department of Learning Disabilities, Coventry and Warwickshire Partnership NHS Foundation Trust, UK; Warwick Medical School, University of Warwick, UK; Department of Brain Sciences, Faculty of Medicine, Imperial College London, UK; Clinical Neuroscience PhD program, Department of Medicine and Surgery, University of Milano-Bicocca, Italy; Warwick Medical School, University of Warwick, UK; Research and Clinical Centre, San Sebastiano Foundation of the Misericordia of Florence, Italy

**Keywords:** Autism spectrum disorder, antidepressants, anti-anxiety medications, systematic review, meta-analysis

## Abstract

**Background:**

Although widely used, the current evidence for the efficacy of antidepressant and anti-anxiety medications for people with autism spectrum disorder (ASD) is limited and conflicting.

**Aims:**

We carried out a systematic review and meta-analysis of randomised controlled trials that assessed the effectiveness of these medications in people with ASD.

**Method:**

We searched the following databases: Cochrane Library, Medline, EMBASE, CINAHL, PsycINFO, ERIC, DARE and ClinicalTrials.gov. Additionally, we hand-searched 11 relevant journals. We used the Cochrane risk-of-bias tool and Jadad score to assess the quality of each included study. We carried out a meta-analysis using a random effects model.

**Results:**

We included 15 randomised controlled trials (13 on antidepressants and two on anti-anxiety medications) for a total of 958 people with ASD. Data showed contradictory findings among the studies, with larger studies mostly showing a non-significant difference in outcomes between the treatment and the placebo groups. Meta-analysis of pooled Yale-Brown Obsessive Compulsive Scale and Clinical Global Impression Scale data from nine studies (60%) did not show any statistically significant inter-group difference on either of the outcome measures. The adverse effects reported were mild and, in most studies, their rates did not show any significant inter-group difference.

**Conclusions:**

Given the methodological flaws in the most included studies and contradictory findings, it is difficult to draw any definitive conclusion about the effectiveness of either antidepressant or anti-anxiety medications to treat either ASD core symptoms or associated behaviours. Robust, large-scale, randomised controlled trials are needed to address this issue.

Autism spectrum disorder (ASD) is a neurodevelopmental disorder that starts in early childhood and often continues into adulthood. The condition is characterised by persistent deficits in social communication and social interaction across multiple contexts, and restricted and repetitive patterns of behaviour (RRB), interests or activities.^1^ The condition affects 1 in every 160 people.^2^

## Psychiatric comorbidities in ASD

ASD is associated with considerable comorbidities with both other neurodevelopmental disorders such as intellectual and developmental disabilities (IDD) and attention-deficit hyperactivity disorder (ADHD), and functional psychiatric disorders such as psychosis, depression and anxiety.^1^ One population-based German study reported depressive disorder in 8.3%, anxiety disorder in 11.1%, psychotic disorder in 1.6% and sleep disorders in 4.9% of 1124 people (aged 0–24 years) with ASD.^3^ In another community-based study of 112 children (aged 10–14 years), the overall rate of anxiety and phobic disorders was reported in 41.9% (social anxiety in 29.2%), ADHD in 28.2% and oppositional defiant disorder or conduct disorder in 30% of the cohort.^4^

## The prevalence of psychotropic medication use in ASD

A systematic review by Jobski et al^5^ included 47 studies of the prevalence of psychotropic medication use in people with ASD (*N* > 300 000) published between 1976 and 2012. The overall median rate of psychotropic use was 45.7%, which was higher among adults (61.5%) than children (41.9%). Polypharmacy of psychotropic use was reported on average among 23% of people with ASD. The most commonly used psychotropics were antipsychotics (median 18.1%), antidepressants (median 17.2%) and psychostimulants (median 16.6%). The use of anti-anxiety medication is reported among 6.8% of children and adolescents.^3^ Psychotropic use in this population seems to have increased over the years.^3,6^ A longitudinal study between 1998 and 2005 found that 57% of adolescents and adults with ASD were taking one or more psychotropic medications at the beginning of the study, which increased to 64% by the end of the study (*P* < 0.05).^7^

## A systematic review of the efficacy of tricyclic antidepressants in ASD

Hurwitz et al^8^ included only two small, crossover randomised controlled trials (RCTs) on clomipramine with 12 children, and 31 children and adults with ASD respectively, in a Cochrane review of the effectiveness of tricyclic antidepressants. One study showed the superiority of clomipramine over placebo and the other study did not show any significant inter-group difference. Another small RCT used tianeptine, which is now withdrawn from the market because of its potential addictive effect. The authors concluded that ‘clinicians considering the use of tricyclic antidepressants need to be aware of the limited and conflicting evidence of effect and the side effect profile when discussing this treatment option with people who have ASD and their carers. Further research is required before tricyclic antidepressants can be recommended for treatment of individuals with ASD’.

## A systematic review of the efficacy of selective serotonin reuptake inhibitors in ASD

Williams et al^9^ published a Cochrane review on the effectiveness of selective serotonin reuptake inhibitors (SSRIs) in people with ASD. They included nine RCTs involving children (*n* = 5) and adults (*n* = 4), assessing the effect of SSRIs on ASD core and associated symptoms: three on fluoxetine, two on fluvoxamine, two on fenfluramine (fenfluramine is withdrawn from the market now) and two on citalopram. Most studies included a small number of participants, but one parallel-design RCT of >100 children showed no superiority of citalopram over placebo. Of the four adult studies, one was based on unpublished data and another included only six participants. The other two small studies (with 30 and 37 participants, respectively) showed significant improvement in some behaviour in the medication groups (one study on fluoxetine and one study on fluvoxamine) compared with the placebo. The authors concluded that ‘there is no evidence of effect of SSRIs in children and emerging evidence of harm. There is limited evidence of the effectiveness of SSRIs in adults from small studies in which risk of bias is unclear’.

## Justification for the current systematic review

No systematic review exists on the efficacy of anti-anxiety medication for people with ASD, although many SSRIs are used to treat anxiety symptoms. One systematic review on the treatment of anxiety in people with ASD found nine studies on cognitive–behaviour therapy and four open-label studies involving fluvoxamine, citalopram and buspirone.^10^ As several studies on antidepressants have been published since the review by Williams et al,^9^ and there is no published systematic review on anti-anxiety medications, we decided to carry out a systematic review and meta-analysis based solely on RCTs (as non-RCTs are likely to produce bias) of antidepressant (both old and new generation) and anti-anxiety (including benzodiazepines, buspirone and beta-blockers) medications for people with ASD for any indication (ASD core symptoms, including communication and language issues; associated behaviours, including agitation and aggression; and/or psychiatric disorders such as depression and anxiety).

## Method

Our objective was to determine the effectiveness of antidepressant and anti-anxiety medications in people with ASD for any indication. The study was registered with International Prospective Register of Systematic Reviews (PROSPERO; identifier CRD42020210708) on 16 October 2020.

### Search strategy

We followed PROSPERO guidelines^11^ and the Preferred Reporting Items for Systematic Review and Meta-Analysis Protocols (PRISMA-P) checklist^12^ to develop our protocol and search strategy. We searched the following databases for English-language publications between January 1985 and October 2020: Medline, EMBASE, PsycINFO, Cochrane Library, CINAHL, ERIC, DARE and ClinicalTrials.gov. Also, we cross-referenced pertinent reviews and articles. We hand-searched for relevant articles published between January 1990 and October 2020 in relevant journals in the field of ASD (*Journal of Autism and Developmental Disorders*, *Autism Research*, *Journal of Autism Spectrum Disorder*), IDD (*Journal of Intellectual Disability Research*, *Journal of Applied Research in Intellectual Disabilities*, *Research in Developmental Disabilities*) and psychopharmacology (*Psychopharmacology*, *Neuropsychopharmacology*, *International Journal of Neuropsychopharmacology*, *Journal of Clinical Psychopharmacology*, *Human Psychopharmacology*, *Journal of Child and Adolescent Psychopharmacology*), using relevant search terms. Search terms are described in Supplementary Appendix 1 available at https://doi.org/10.1192/bjo.2021.1003, and included terms like RCTs, depression, anxiety and anxiety disorder.

### Selection criteria

The titles, abstracts and full papers were screened independently by two authors (M.R. and J.S.), using pre-piloted eligibility criteria (Supplementary Appendix 2) designed as per Cochrane Library^13^ and PROSPERO^11^ guidelines. All RCTs in ASD involving antidepressants and anti-anxiety medications were included. The list of excluded studies with reasons for exclusion is provided in Supplementary Appendix 3.

### Participants

Participants had a diagnosis of an ASD, defined using standardised criteria such as the DSM or ICD, or based on a clinical assessment. The diagnosis may or may not have been made with a standardised diagnostic instrument. No age limit was applied. Studies that included people with IDD were included if the participants also had a confirmed diagnosis of ASD. We excluded any RCT that included less than ten participants, in line with our previous reviews.^14–17^

### Intervention

We included studies of both new- and old-generation antidepressants, including SSRIs, serotonin–noradrenaline reuptake inhibitor, tricyclics, any other types of antidepressants and any type of anti-anxiety medications such as benzodiazepines, buspirone, beta-blockers and pregabalin, regardless of dosage used or frequency of administration.

### Design and comparators

Only RCTs that evaluated the effectiveness of antidepressants and anti-anxiety medications on people with ASD of any age (including children, adolescents and adults) were included in this review. The control treatment could be a placebo or another medication.

### Outcome measures

Any standardised validated outcome measure to assess mental state, including symptoms of any psychiatric illness such as depression and anxiety; ASD core symptoms such as language and communication impairment and RRB; and any other associated behaviours such as agitation, aggression, irritability, hyperactivity and any other problem behaviour.

### Selection process

Any discrepancy in scoring was resolved by discussion and, if necessary, arbitration by a third author (S.D.). Data were organised with Mendeley (Version 1.19.8 for Windows, Elsevier, Amsterdam, the Netherlands; see https://www.mendeley.com/download-desktop-new/)Reference Manager.^18^

### Data extraction

Data were extracted independently by two authors (R.L. and M.M.), using a proforma modified from the Cochrane template^19^ (Supplementary Appendix 4). As people with ASD are likely to be more sensitive to psychotropic medication, we have taken note of the adverse effects described in the included studies^20–34^ and presented the findings in Table 1.

**Table 1:** Summary findings

Study author and date	Study type, (methods used for ASD diagnosis)	Dose of medications	Participants (N, Age (mean ± s.d.), gender, IQ (mean ± s.d.), Intervention, FU	Outcome measures used	Findings, Jadad score	Comments
Agomelatine						
Ballester et al, 2019^20^	Crossover (DSM-V)	Agomelatine (25 mg/day)	*N* = 23 Age: 18–65 years (53 ± 12 years) Male: 83% IQ: 100% IDD Agomelatine/Placebo: 23 FU: 1 month	TST, TiB, SoL, Number of awakenings during TiB, Wake after sleep onset, Sleep efficiency	Only night TST significantly increased (mean 83 min) during agomelatine treatment (abnormal TST among 50% participants pre-treatment *v*. 16% post agomelatine treatment) (*P* = 0.016). No significant change in other sleep parameters. The authors reported only mild and transient adverse events associated with the agomelatine treatment. Jadad = 4	Small sample size risking type II error. The outcome is not directly related to ASD core symptoms, although sleep problem is common in ASD. Only one sleep parameter has shown improvement.
Buspirone						
Chugani et al, 2016^21^	Parallel design (DSM-IV, ADI-R and ADOS)	Buspirone 2.5 mg twice daily *v*. Buspirone 5 mg twice daily	*N* = 166 (142 completed) Age: 2–6 years Male: 82.5% IQ: Not reported Buspirone 2.5 mg twice daily = 54 Buspirone 5 mg twice daily = 55 Placebo = 57 FU: 24 weeks	ADOS-CTS, ADOS-SA and ADOS-RRB, ABC, VABS, RBS, SPS, C-YBOCS-PDD, CGI, Leiter Parent-Report	A non-significant intergroup difference in the primary outcome ADOS-CTS. RRB score as per CYBOCS-PDD showed a significant difference from baseline to FU (*P* = 0.03) in 2.5 mg twice daily dose but not in the 5 mg twice daily dose or the placebo group. There was no significant intergroup difference in the rate of adverse events. Jadad = 5	Reasonable sample size but contradictory findings based on different dosages and outcome measures.
Ghanizadeh & Ayoobzadehshirazi, 2015^22^	Parallel design (Clinical diagnosis)	Buspirone 10 mg/day (<40 kg) or 20 mg/day (>40 kg)	*N* = 40 (34 completed) Age: Mean: 7 years Male: 82.5% IQ: Not reported Buspirone: 16 Placebo: 18 FU: 4 and 8 weeks	ABC-Irritability subscale	Thirteen (81.2%) in the buspirone group and seven (38.9%) in the placebo group showed a >30% decline in irritability score, although total irritability score improved significantly from the baseline score in both groups (both *P* < 0.001). No major adverse events are reported. The most common adverse events associated with buspirone treatment were increased appetite (61%), drowsiness (11%), and fatigue (11%). Jadad = 5	Short study duration. Small sample size risking a type II error.
Citalopram King et al, 2009^23^	Parallel design (DSM-IV-TR, ADI-R, ADOS)	Citalopram 10 mg/5 mL, 2.5 mg-20 mg/day	*N* = 149 (123 completed) Age: 5–17 years (9.4 ± 3.1 years) Male: 86% IQ: 40% had a non-verbal IQ <70 Citalopram: 60 Placebo: 63 FU: 12 weeks	C-YBOCS-PDD total score + RRB, CGI-I, RBS, ABC	No significant intergroup difference in C-YBOCS-PDD, RRB, and CGI-I scores. Citalopram group showed more adverse effects such as increased energy, impulsiveness, decreased concentration, hyperactivity, stereotypy, diarrhoea, insomnia, and dry skin or pruritus. Jadad = 5	A large sample is likely to have given adequate power to the study but the overall dropout rate maybe a bit high. However, an ITT analysis should have mitigated this.

Clomipramine Gordon et al, 1993^24^	Crossover (DSM-III-R, ADI)	Clomipramine *v*. desipramine 25 mg/day-250 mg/day (or 5 mg/kg/day)	*N* = 28 (24 completed) Age: 6–23 years (10.4 ± 4.1 years) Male: 62.5% IQ: 30–107 (*n* = 19; 79.16% ≤70) Clomipramine/Placebo: 12 Clomipramine/Desipramine: 12 FU: week 5	Modified CPRS OCD subscale, CGI	Clomipramine was superior to both placebo (*P* ≤ 0.001) and desipramine (*P* ≤ 0.005) in improving CPRS subscales and CGI scores. There was no effect of age, gender, IQ level, and dose of clomipramine on the outcome. The authors reported minor adverse effects in all three groups without any significant intergroup difference. Jadad = 3	Small sample size risking a type II error. A short washout period may have caused a carry-over effect on long-standing behaviours.
Remington et al, 2001^25^	Crossover Latin square (DSM-IV)	Clomipramine (100–150 mg/day, mean: 128.4 mg/day); Haloperidol (1–1.5 mg/day; mean: 1.3 mg/day)	*N* = 36 Age: 10–36 years, (mean: 16.3) Male: 86% IQ: Not stated Clomipramine/ Haloperidol/ Placebo: 36 FU: 7 weeks	Global ASD measures CARS, ESRS, DOTES, ABC- subscales	Clomipramine did not show any superiority over placebo, but haloperidol did (*P* < 0.05) on the global measure of autistic symptoms, and ABC-irritability, hyperactivity, and stereotype subscale scores. Adverse events associated with clomipramine included fatigue (*n* = 4), tremors (*n* = 2), tachycardia (*n* = 1), insomnia (*n* = 1), diaphoresis (*n* = 1), nausea or vomiting (*n* = 1), and decreased appetite (*n* = 1). Four of them also showed problem behaviour. Jadad = 4	Small sample size risking a type II error. A short washout period may have caused a carry-over effect on long-standing behaviours. A complicated study design has made the interpretation of findings difficult.
Fluoxetine						
Herscu et al, 2020^26^ (SOFIA study)	Parallel design (DSM-IV-TR)	Fluoxetine 2–18 mg/day (Mean: 11.8 mg/day)	*N* = 158 (121 completed) Age: 5–17 years Male: 85.5% IQ: Not reported Fluoxetine: 56 Placebo: 65 FU: 14 weeks	CYBOCS-PDD total + RRB score, CGI, CSQ	No significant intergroup difference in any of the outcome measures. ‘Much improved’ and ‘very much improved’ in 23% of fluoxetine and 34% placebo group as per CGI-I scores. The rate of adverse effects was similar in the two groups and both groups showed a high rate of activation (fluoxetine: 42%, placebo: 45%). Jadad = 5	A large sample may have given adequate power, but the authors suggested that a low starting dose may have prevented a therapeutic effect.
Hollander et al, 2005^27^	Crossover (DSM-IV, ADI-R, ADOS)	Fluoxetine 2.5 mg/day to 0.8 mg/kg/day	*N* = 45 (39 completed) Age: 5–17 years Male: 76.9% IQ: 30–132 (63.65 ± 27.9) Fluoxetine/Placebo: 39 FU: 20 weeks	C-YBOCS RRB score, Autism global symptoms, CGI	Moderate to large effect size on C-YBOCS RRB score (z = −2.075, SE = 0.407, *P* = 0.038). No effect on CGI, speech, social interaction. There was no significant intergroup difference in the rate of adverse effects. The fluoxetine group reported a numerically lower rate of insomnia, anxiety, urinary incontinence, and mild weight gain but a higher rate of sedation, agitation, diarrhoea, and anorexia when compared with the placebo group. Jadad = 3	Small sample size risking a type II error. A short washout period may have caused a carry-over effect on long-standing behaviours.
Hollander et al, 2012^28^	Parallel design (DSM-IV, ADI-R, ADOS)	Fluoxetine 10 mg/day starting dose to maximum of 80 mg/day	*N* = 37 (34 completed) Age: 18–60 years (34.31 ± 14.26 years) Male: 69% IQ: 30–161 (103.25 ± 28.45) Fluoxetine: 21 Placebo: 13 FU: 12 weeks	YBOCS RRB score, CGI, ABC, HAM-D	Fluoxetine was superior to placebo on YBOCS RRB score (F = 9.24, df = 1, 30.7, *P* = 0.005, d = 0.53). CGI improvement in obsessive-compulsive symptoms (score 2 or less): 50% in the fluoxetine group *v*. 8% in the placebo group (*P* = 0.03). HAM-D scores were well under the threshold for a diagnosis of depressive disorder in both groups. A statistical comparison of intergroup adverse effects was not possible. In the fluoxetine group, 1.4 adverse effects per patient were reported compared with 0.6 in the placebo group. Adverse events associated with the fluoxetine treatment were mild to moderate and included bad dreams (*n* = 3), mild insomnia (*n* = 3), mild dry mouth (*n* = 3), and headaches (*n* = 3). There was no statistically significant intergroup difference in the rates of suicidal ideation between the fluoxetine (6%) and the placebo group (0%) (*P* = 1.00). No one in either group reported suicidal gestures or attempts. Jadad = 3	Small sample size risking a type II error.
Reddihough et al, 2019^29^	Multicentre, parallel design (DSM-IV-TR, ADI-R)	Fluoxetine: commencing at 4 or 8 mg/day for the first week (4 mg if <40 kg; 8 mg if ≥40 kg) maximum dose was 20 mg/day (participants <40 kg) or 30 mg/day (participants ≥40 kg).	*N* = 146 (109 completed) Age: 7.5–18 years (11.2 ± 2.9 years) Male: 80% IQ: 30% IDD Fluoxetine: 54 Placebo: 55 FU: 16 weeks	C-YBOCS-PDD, RBS, ABC, CGI	The mean C-YBOCS-PDD score decreased in the fluoxetine group by 3.72 points (95% CI, −4.85 to −2.60) and in the placebo group by 2.53 points (95% CI, −3.86 to −1.19). The between-group mean difference at follow up was −2.01 (95% CI, −3.77 to −0.25; *P* = 0.03) (adjusted for stratification factors), and in the prespecified model with further adjustment, it was −1.17 (95% CI, −3.01 to 0.67; *P* = 0.21). The multiple imputation analysis did not show any statistically significant intergroup difference (mean difference, −1.82; 95%CI, −3.71 to 0.06; *P* = 0.06). The rate of commonly observed adverse effects such as irritability, mood disturbance, nausea, vomiting, and sleep disorders did not show any significant intergroup difference (fluoxetine, 45%, the total number of events 2.5 (s.d. ± 1.6) and placebo, 48%, the total number of events 2.6 (s.d. ± 2.1). Jadad = 4	Initial analysis showed the superiority of fluoxetine over placebo, but this difference was not maintained when baseline imbalance in outcome scores between two arms was adjusted for. Also, the confidence interval of the scores included a minimally clinically important difference. High dropout rate (25%)
Fluvoxamine						
McDougle et al, 1996^30^	Parallel design (DSM-III-R, ICD-10)	Fluvoxamine: 50 mg/day, increased every 3 or 4 days to a maximum of 300 mg/day as tolerated	*N* = 30 (all completed) Age: 18–53 years (30.1 ± 7.7 years) Male: 90% IQ: 25–114 (79.9 ± 29.7) Fluvoxamine: 15 Placebo:15 FU: 12 weeks	YBOCS, CGI, VABS maladaptive behavior subscale, Brown Aggression Scale, Ritvo-Freeman Real-Life Rating Scale	A significantly higher proportion of the fluvoxamine group (53%) was reported to be responders to treatment compared with the placebo group (0%). Fluvoxamine was superior in improving repetitive thoughts and behaviour (*P* < 0.001), problem behaviour (*P* < 0.001), aggression (*P* < 0.03), some aspects of social relatedness (*P* < 0.04), and language usage (*P* < 0.008). Age, the severity of autism symptoms, and the level of IQ did not influence the treatment response. Other than mild sedation and nausea in a few cases, fluvoxamine was well tolerated. No one reported dyskinesias, seizures, electrocardiograph change, anticholinergic, or adverse cardiovascular events. Jadad = 3	Small sample size risking a type II error.
Sugie et al, 2005^31^	A crossover study in the context of genetic evaluation (DSM-IV)	Fluvoxamine 1–3 mg/kg body weight/day	*N* = 19 (18 completed) Age: 3 years - 8 years 5 months (mean: 5 years 4 months) Male: 79% IQ: Not reported Fluvoxamine/Placebo: 18 FU: 12 weeks	BAS, CGI	Fluvoxamine showed a statistically significant improvement in two (flighty eye movements and delayed or peculiar or inappropriate speech) of the 20 BAS items compared with the placebo. Fluvoxamine did not show any significant adverse effects other than transient nausea and hyperactivity. Jadad = 5	Small sample size risking a type II error. No adjustment is made for multiple comparisons to avoid any type I error.
Sertraline						
Potter et al, 2019^32^	Parallel design (DSM-IV, ADOS)	Sertraline (20 mg/mL), under four years: 2.5 mg/day (0.125 mL); four years or older: 5 mg/day (0.25 mL).	*N* = 58 (45 completed) Age: 2–6 years Male: 79.5% IQ: (DQ: sertraline: 48.72 ± 28.46; placebo: 50.63 ± 24.05) Sertraline: 24 Placebo: 21 FU: 6 months	MSEL (expressive language raw score and age equivalent combined score), CGI, PVET, VABS-II, ABC-C, PAS-R, SRS, SPM-P, PLS-5, Visual Analog Scale	ITT analysis did not show any significant intergroup difference in any outcome measure. No statistically significant intergroup difference in the rate of adverse effects. No sertraline-associated serious adverse event was reported. Jadad = 4	Small sample size risking a type II error.
Venlafaxine						
Carminati et al, 2016^33^	Parallel design (ICD-10)	Venlafaxine 18.75 mg/day Zuclopenthixol or clonazepam introduced or continued with dose adjustment as necessary	*N* = 13 Age : venlafaxine : 18–30 years (median : 22 years); placebo: 19–32 years (median : 19 years) Male: 84.5% IQ: 100% IDD Venlafaxine: 6 Placebo: 7 FU: 8 weeks	ABC, BPI, CGI Reduction in usual doses of zuclopenthixol or clonazepam, Simpson-Angus Rating Scale	Univariate analyses showed that the symptom of irritability improved in the entire sample (*P* = 0.061), although no difference was observed between the venlafaxine and the placebo group. No significant decrease in hyperactivity/noncompliance was observed during the study. Global improvement was observed in 33% of participants treated with venlafaxine and in 71% of participants in the placebo group (*P* = 0.29). Decreased cumulative doses of clonazepam and zuclopenthixol were required for the venlafaxine group. Multivariate analyses (principal component analyses) with at least three combinations of variables showed that the two populations could be clearly separated (*P* < 0.05). A few adverse effects in the venlafaxine group included excessive salivation, slight elbow stiffness, mild finger tremor, and head dropping. No comparative data for the placebo group is provided by the authors. Jadad = 3	Small sample size. Very complicated design involving the use of additional medications, which makes interpretation of the findings difficult. Different results are shown depending on the statistical analysis used (univariate *v*. multivariate).
Niederhofer, 2004^34^	Crossover (ICD-10)	Venlafaxine: 30 mg/day	*N* = 15 (14 completed) Age: 5.2–11.7 years (7.1 ± 3 years) Male: 100% IQ: 55-79 (67 ± 12) Venlafaxine/Placebo: 14 FU: 6 weeks	CGAS, CGI, ABC, Modified CPRS	No statistically significant intergroup difference in the clinician's rating of videotaped behaviour according to CPRS, CGAS, and CGI. Statistically significant better improvement in ABC sub scores of irritability (*P* = 0.04), inadequate eye contact (*P* = 0.042), hyperactivity (*P* = 0.035), and lethargy (*P* = 0.043) according to the teacher's rating during the venlafaxine treatment compared with the placebo phase. Adverse effects such as increased thirst, drowsiness, sleep disturbance, sadness, dizziness, irritability, decreased activity have been reported in the venlafaxine treatment group but without providing the number of participants affected or any comparative data in the placebo phase. Jadad = 3	Very small all-male clinic sample, so the generalisability of the findings is questionable.

ABC, Aberrant Behaviour Checklist; ABC-C, Aberrant Behavior Checklist-Community version; ADI-R, Autism Diagnostic Interview-Revised; ADOS-CTS, Autism Diagnostic Observation Schedule-Composite Total Score; ADOS-SA, Autism Diagnostic Observation Schedule-Social Affect; ADOS-RRB, Autism Diagnostic Observation Schedule-Restricted and Repetitive Behaviour; ASD, Autism Spectrum Disorder; BAS, Behaviour Assessment Scale; BPI, Behaviour Problems Inventory; CARS, Childhood Autism Rating Scale; CGAS, Children's Global Assessment Scale; CGI, Clinical Global Impressions Scale; CGI-I, Clinical Global Impressions-Improvement Scale; CI, Confidence Interval, CPRS OCD subscale, Comprehensive Psychological Rating Scale Obsessive Behaviour Disorder subscale; CSQ, Caregiver Strain Questionnaire; C-YBOCS-PDD, Children's Yale-Brown Obsessive Compulsive Scale-Modified for Pervasive Developmental Disorders; df, degree of freedom; DOTES, Dosage Treatment Emergent Symptom Scale; DSM-V, Diagnostic and Statistical Manual of Mental Disorders, 5th Edition; DSM-IV, Diagnostic and Statistical Manual of Mental Disorders, 4th Edition; DSM-IV-TR, Diagnostic and Statistical Manual of Mental Disorders, 4th Edition Text Revision; DSM-III-R, Diagnostic and Statistical Manual of Mental Disorders, 3rd Edition-Revised; DQ, Development Quotient; ESRS, Extrapyramidal Symptom Rating Scale; FU, Follow-up; ICD-10, International Classification of Diseases 10th Revision; HAM-D, Hamilton Depression Rating Scale; IDD, Intellectual and Developmental Disability; ITT, Intention to Treat; IQ, Intelligence Quotient; MSEL, Mullen Scales of Early Learning; N, number; PAS-R, Preschool Anxiety Scale-Revised; PLS-5, Preschool Language Scales, 5th Edition; PVET, Passive-Viewing Eye Tracking Task; RBS, Repetitive Behavior Scale; RRB, Restrictive Repetitive Behaviors; SD, Standard deviation; SE: Standard Error; SoL, Sleep onset Latency; SPM-P, Sensory Processing Measure-Preschool; SPS, Sensory Profile Scale; SRS, Social Responsiveness Scale; TiB, Time in Bed; TST, Total Sleep Time; VABS-II, Vineland-Adaptive Behaviour Scale-II (maladaptive behaviors); YBOCS, Yale-Brown Obsessive Compulsive Scale.

### Quality assessment of included studies

The quality of the included RCTs was assessed independently by two authors (R.L. and M.M.), using the Cochrane risk-of-bias tool^35^ (Supplementary Appendix 5) and the Jadad score.^36^ Any discrepancy in scores was resolved by discussion between the authors, with arbitration from a third author (B.L.). Overall findings were presented in a narrative format, but a meta-analysis was also carried out where possible.

### Data synthesis

A narrative synthesis of the findings is provided in Table 1.^20–34^ Where there were more than one studies, a meta-analysis was carried out. A random-effects odds ratio or standardised mean difference with 95% confidence interval meta-analysis was performed, depending on the type of data gathered. Heterogeneity was tested with the *χ*^2^-test and *I*^2^-statistic test of heterogeneity. If there was substantial heterogeneity (*I*^2^ > 50%) as per the Cochrane guide, a further sensitivity analysis was carried out.^37^

### Meta-analysis

We pooled data for meta-analysis only from those RCTs that presented data with the same outcome measure. A standardised mean difference was calculated for those studies that presented means and s.d. for scores based on an outcome measure in the two groups.^37^ RevMan version 5.3 (The Nordic Cochrane Centre, The Cochrane Collaboration, Copenhagen, Denmark; see www.training.cochrane.org) software for Windows 10 was used for random-effects meta-analysis, because of the heterogeneity.

### Meta-bias(es)

Funnel plots^38^ were drawn, and an Egger's value was calculated^39^ to assess publication bias (Supplementary Appendices 6 and7).

### Confidence in cumulative estimate

The quality of evidence was assessed across the risk-of-bias domains of consistency, directness, precision and publication bias. Any ambiguous studies that were deemed of low quality were excluded from the review. The PRISMA-P checklist was completed, and the overall quality of the systematic review and the meta-analysis was assessed with the A Measurement Tool to Assess Systematic Reviews 2 (AMSTAR 2) checklist (Supplementary Appendix 8).^40^ No ethical approval was necessary for this review as no individual patient-related data were collected or analysed.

## Results

### Search findings

**Fig. 1:**
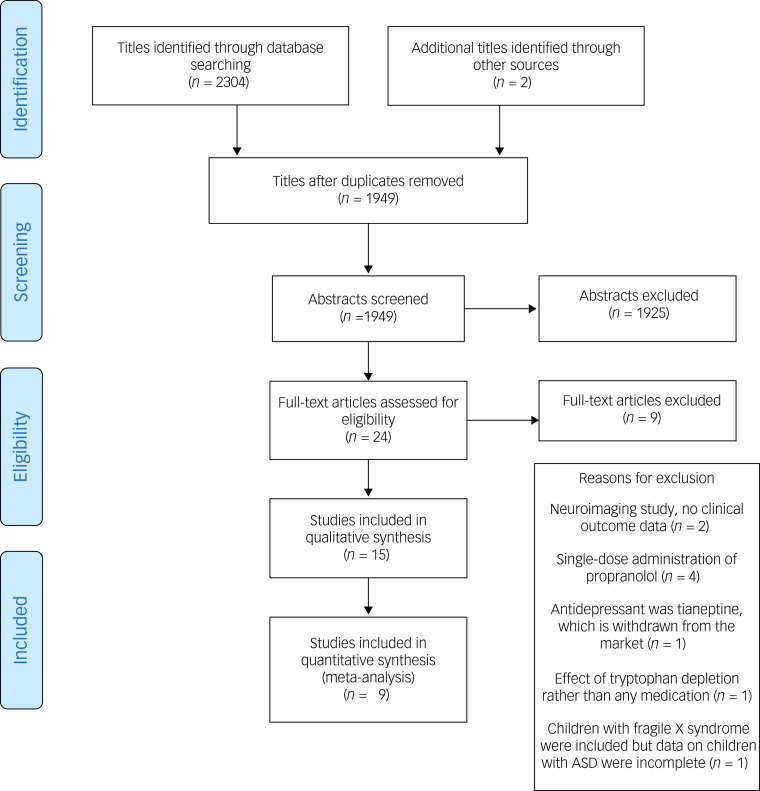
Preferred Reporting Items for Systematic Review and Meta-Analysis (PRISMA) flow chart of paper selection. ASD, autism spectrum disorder.

Initially, we identified 2306 titles from the literature search and hand search of the relevant journals, and ultimately included 15 studies in this review (see Fig. 1). Data were first extracted on the 10 December 2020. We included 13 studies on antidepressants (four on fluoxetine, two on clomipramine, two on fluvoxamine, two on venlafaxine, one on citalopram, one on sertraline and one on agomelatine) and two on the anti-anxiety medication buspirone. We did not find any RCT on the long-term use of beta-blockers or benzodiazepines.

### Description of the study population

These studies included 958 participants in total. There was a male preponderance among the participants, reflecting the male preponderance among people with ASD. Seven RCTs (53.85%) on antidepressants included children and adolescents only (age ranged from 2 to 18 years), four RCTs (30.76%) included adults only and another two RCTs (15.38%) included both children and adults (age ranged from 6 to 36 years). All participants (adults) in two studies (15.38%) had IDD, in eight studies (61.53%) a high proportion had IDD and IQ was not reported in three studies (23.07%). Both RCTs on anti-anxiety medications included children and adolescents only (age starting from 2 years), and none reported participant IQ. Information on the inclusion and exclusion criteria for each study, along with comorbidities and funding source, is provided in Supplementary Appendix 9.

### Outcome measures used in the included studies

We did not find any RCT that used outcome measures for anxiety and depression. Instead, these RCTs assessed the effect of the medications on ASD core symptoms such as RRB, and other associated behaviours such as irritability, hyperactivity and aggression. The two most common outcome measures used were the ASD core symptom of RRB measured by the Yale-Brown Obsessive Compulsive Scale (YBOCS)^41^ and Children-YBOCS (C-YBOCS),^42^ and the Clinical Global Impression-Improvement (CGI-I) Scale (National Institute of Mental Health).^43^ Both scales were used in seven RCTs (46.66%). Some of the other outcome measures included the Aberrant Behaviour Checklist-Community,^44^ Repetitive Behaviours Scale,^45^ Children's Global Assessment Scale^46^ and modified Children's Psychiatric Rating Scale.^47^

### A narrative synthesis of the data

#### Old generation of antidepressants

##### Clomipramine

The findings of the included studies are summarised in Table 1.^20–34^ Two RCTs on old-generation antidepressant tricyclics were on clomipramine, both of which were small crossover trials (*n* = 24 and 36);^24,25^ one compared clomipramine with desipramine and placebo,^24^ and the other with haloperidol and placebo.^25^ One found clomipramine to be marginally significantly better than placebo in treating ASD core symptoms like RRB,^24^ but the other one did not show any significant inter-group difference.^25^

#### New generation of antidepressants, including SSRIs

##### Fluoxetine

Among the new generation of antidepressants, most RCTs were on SSRIs. Four RCTs were on fluoxetine, one of which was a small crossover trial of 45 children and the rest were parallel-design RCTs involving 158 children, 146 children and 37 adults, respectively.^26–29^ Of the two larger RCTs, one^26^ showed no statistically significant inter-group difference on RRB, but the authors felt the overly cautious dosing regime and the short follow-up may have prevented the therapeutic effect of fluoxetine. The other large parallel-design RCT^29^ initially showed a significantly better outcome of fluoxetine compared with the placebo, but the study evidenced a high drop-out rate (25%), the significant difference was not sustained when baseline imbalance between the two groups was adjusted for, and the confidence interval of the difference in the outcome score included minimal clinically effective difference. The smaller crossover RCT^27^ and parallel-design^28^ RCT showed a significantly better efficacy of fluoxetine over placebo.

##### Fluvoxamine

In two small trials on fluvoxamine, one involved 19 children in a crossover trial^31^ and the other involved 30 adults in a parallel-design RCT.^30^ The parallel-design study^30^ showed a statistically significant better effect of fluvoxamine on RRB and social communication and aggression. The crossover study^31^ was carried out in the context of a genetic study, which showed a significant improvement in two of the 20 items (10%) in a behaviour scale devised by the authors in the fluoxetine compared with the placebo phase.

##### Citalopram

One large-scale, parallel-design RCT involving 149 children^23^ did not show any significant inter-group difference between the citalopram and the placebo group in RRB and CGI-I score.

##### Venlafaxine

There were two small RCTs involving venlafaxine: one included 14 male children in a crossover study^34^ and found conflicting evidence depending on the outcome measure used, and another study included^33^13 children in a parallel-design RCT and did not find any significant inter-group difference in efficacy.

##### Sertraline

A parallel-design study involving 58 children did not show any significant inter-group difference in the efficacy between sertraline and the placebo.^32^

##### Agomelatine

A crossover trial of 23 adults showed improvement in night total sleep time but no other sleep parameters during agomelatine treatment, but not in the placebo phase.^20^ No ASD core symptoms were assessed in this study.

We did not find any RCT involving paroxetine, escitalopram, mirtazapine, duloxetine or vortioxetine.

#### Anti-anxiety medication

##### Buspirone

We found two parallel-design RCTs on buspirone, involving 40 and 166 children.^21,22^ The larger study^21^ did not show any significant inter-group difference in the primary outcome measure of Autism Diagnostic Observation Schedule composite score^48^ at the 24-week follow-up. For RRB, 5 mg twice daily dose and placebo did not show any significant change from baseline, but 2.5 mg twice daily dose showed a significant improvement. The smaller study^22^ showed a significant improvement in the Aberrant Behaviour Checklist irritability subscale score^44^ in the buspirone compared with the placebo group.

### Adverse effects

In most studies, there was no statistically significant inter-group difference in the rate of medication-related adverse events, except for a large study of citalopram^23^ and a small study of clomipramine (see Table 1).^25^ In the large study, citalopram was more significantly associated with adverse events, such as hyperactivity, decreased concentration, impulsiveness and stereotype, compared with the placebo.^23^ In the other study, clomipramine^25^ was more significantly associated with problem behaviour, fatigue, tremor and cardiac problems, compared with the placebo.

### Quality of the included papers

**Fig. 2:**
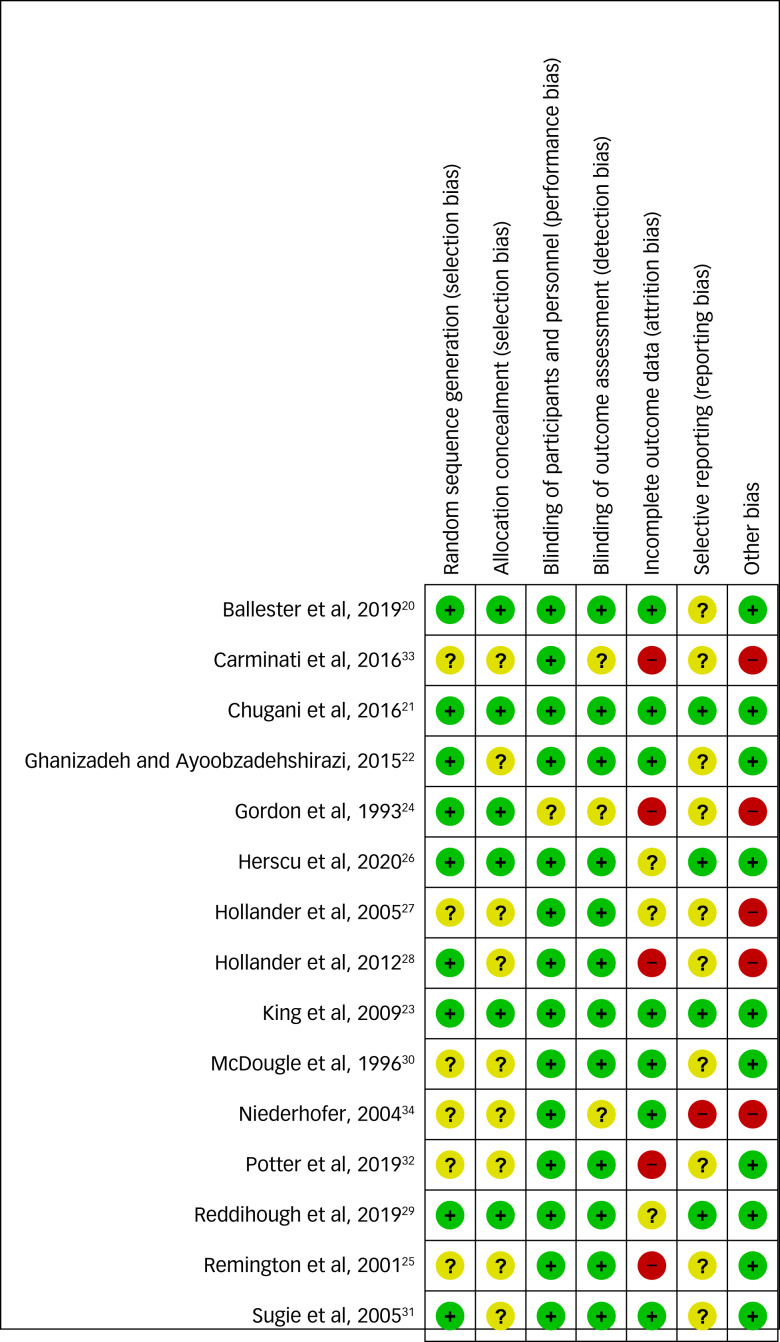
Cochrane risk-of-bias summary scores.^20–34^

The Cochrane risk-of-bias analysis showed seven out of 15 studies (46.66%) scored a high risk in at least one item, of whom four (26.66%) showed a high risk in two items (see Fig. 2). Ten out of 15 studies (66.66%) received a Jaded score of <5.

### Meta-analysis (RRB and CGI scores)

**Fig. 3:**
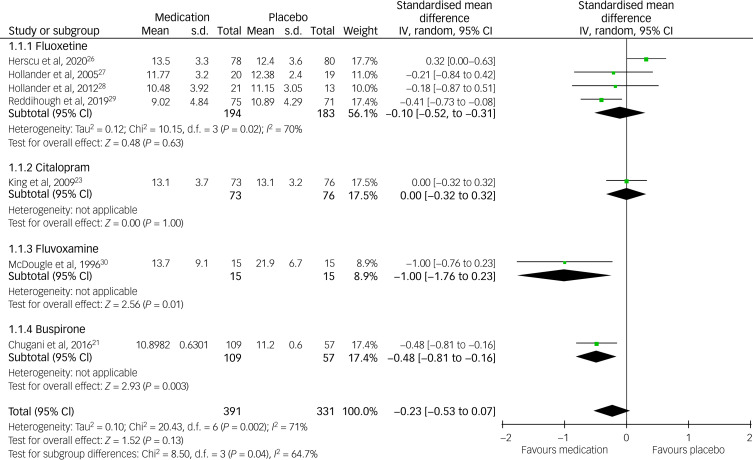
Forest plot based on the Children-Yale-Brown Obsessive Compulsive Scale (Y-BOCS) pooled data.

**Fig. 4:**
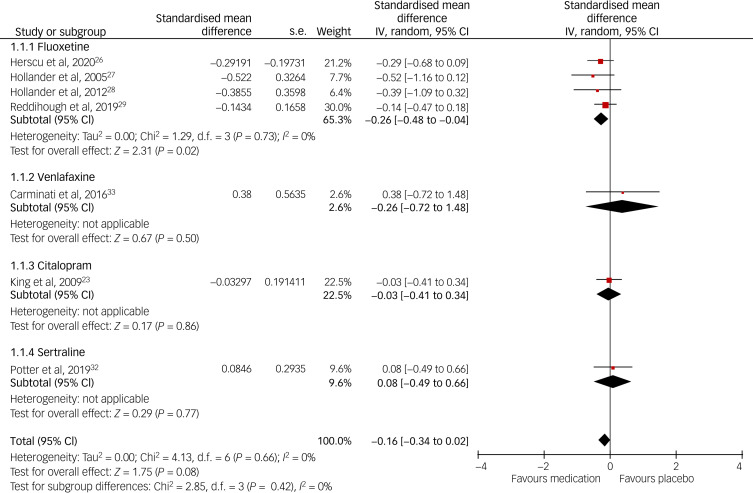
Forest plot based on the Clinical Global Impression-Improvement Scale (CGI-I) pooled data.

Using YBOCS-/C-YBOCS-based RRB scores, we managed to pool data for meta-analysis from seven (46.66%) out of 15 RCTs (four on fluoxetine, one on fluvoxamine, one on citalopram and one on buspirone) (see Fig. 3). Similarly, using the CGI-I score, we were able to pool data from seven RCTs, all of which were on antidepressants (46.66%) (four on fluoxetine, one on citalopram, one on sertraline and one on venlafaxine) (see Fig. 4). Altogether, it was possible to pool data from nine (60%) RCTs. Fluoxetine and citalopram did not show any significant inter-group difference, but the fluvoxamine score was significantly better than the placebo. Buspirone data could not be pooled together as there was only one study that used RRB as an outcome, which showed a significantly better effect of the medication when the two different doses were combined (i.e. 5 mg and 2.5 mg twice daily dose, respectively). The overall pooled data on antidepressants and anti-anxiety medication did not show any statistically significant inter-group difference, although the heterogeneity was high at *I*^2^ = 71 (see Fig. 3). Neither the individual antidepressants nor the pooled data on CGI-I showed any statistically significant inter-group difference, and the heterogeneity was low at *I*^2^ = 0 (see Fig. 4). Funnel plots did not show any publication bias for either forest plot, and Egger's test scores were *P* = 0.42 for the YBOCS and *P* = 0.87 for the CGI-I forest plot, respectively. When the studies with a high risk of bias were removed from the meta-analysis, the medication group did not show any significant difference from the placebo group.

## Discussion

### Overall findings

Our systematic review found 13 RCTs involving antidepressants clomipramine, fluvoxamine, fluoxetine, citalopram, sertraline, venlafaxine and agomelatine, and two on the anti-anxiety medication buspirone. The evidence for the efficacy of old-generation tricyclic antidepressants in improving ASD symptoms and associated behaviour is lacking, based on only two small crossover trials with contradictory results. Tricyclics are not recommended for use because of their anticholinergic and cardiac adverse effects, and the risk of fatality associated with overdose. As for the new generation of antidepressants (SSRIs and serotonin–noradrenaline reuptake inhibitors), the evidence is non-conclusive. Only three studies included >100 participants (158 and 146 children for fluoxetine, and 149 children for citalopram respectively). The citalopram study did not show a statistically significant inter-group difference. Of the two large fluoxetine studies, one did not show any superiority over placebo, and although the other one showed the superiority of fluoxetine in the initial analysis, this difference disappeared when adjusted for baseline inter-group imbalance and the confidence intervals were taken into account. This study also had a high drop-out rate.

The rest of the smaller studies showed contradictory results, with some showing superiority of the medication over placebo and the others not. Only one of the two RCTs on buspirone included >100 participants (166 children), which did not show any inter-group difference in the primary outcome of Autism Diagnostic Observation Schedule-Composite Score but showed a statistically significant improvement in RRB only with the smaller daily dose (2.5 mg twice daily), but not the higher dose (5 mg twice daily). No RCT was found on any other anti-anxiety medications such as benzodiazepines or beta-blockers (the only RCTs on beta-blockers were on a single-dose administration; see Supplementary Appendix 3). Benzodiazepines are not recommended for long-term use because of their effect on cognition, paradoxical emergence of aggression, tolerance and addiction. Similarly, the administration of high doses of beta-blockers risks adverse effects such as lowering of blood pressure and respiratory failure.

Meta-analysis showed no statistically significant inter-group difference between antidepressants and the placebo. These findings are consistent with the previous Cochrane review.^9^ However, we included additional RCTs that were not included in the previous Cochrane review.^9^

We have also carried out a systematic review on the RCTs of anti-anxiety medication on people with ASD, which was not done before.

### Methodological issues

Most studies were on children, but some included data on adults with ASD, and no specific age effect was observable from the data presented in Table 1.^20–34^ Of the 15 RCTs, six (40%) used a crossover design. Crossover design may not be ideal to assess long-standing behaviour outcomes, particularly if the washout period is relatively short, as is the case in the included studies. Several studies recruited participants with ASD who also had IDD, but often did not present data separately on those participants. Therefore, it is difficult to assess the influence of IQ on the outcome.

The overall quantity and quality of the RCTs are, at best, poor. However, our meta-analysis mitigates that to some extent. Based on the currently available weak and contradictory evidence, our systematic review can neither support nor refute the use of antidepressant and anti-anxiety medications for the treatment of ASD core symptoms such as RRB, impaired social communication or associated behaviour such as aggression, irritability and agitation, despite their widespread use in this population. However, this systematic review does not answer the question of whether antidepressants and anti-anxiety medications are effective in treating depression and anxiety in people with ASD, as we did not find any RCTs involving these medications for the treatment of depression and anxiety in the ASD population. In fact, most included RCTs excluded participants with a comorbid psychiatric disorder (see Supplementary Appendix 9). Therefore, the evidence for this has to be extrapolated from studies done on individuals without ASD. Although the RCTs included in our review did not show any significant inter-group difference in the rate of adverse effects in most studies, these medications have known adverse effects. Therefore, the clinicians considering the use of these medications for people with ASD, particularly to treat ASD core symptoms and associated behaviours, should carefully weigh up the supporting evidence (or lack of it) and the potential for adverse effects, which may be more pronounced in people with ASD.

Information on funding was not available for three studies, but the available data shows that apart from possibly one exception, the rest of the studies were not funded by any pharmaceutical company (see Supplementary Appendix 9). Therefore, the findings from these studies could be considered independent and not influenced by the funding organisations.

### Weakness

Several drawbacks have to be considered when interpreting the data of this systematic review. Different studies used different outcome measures, which produced heterogeneity when the findings were combined. As a result, we could only pool data for meta-analysis from those studies that used the same outcome measure, such as the YBOCS/C-YOBCS and CGI-I, which were reported in only nine (60%) of the 15 RCTs. Although the meta-analysis of CGI-I data showed no heterogeneity, the forest plot involving data from YBOCS/C-YBOCS scores showed high heterogeneity. Another major problem is the small sample size in most studies. For example, only four studies (26.7%) included >100 participants. This makes the findings from most of these studies difficult to generalise. Almost half of the studies have also shown at least one area of a high risk of bias according to the Cochrane risk-of-bias assessment.

### Strengths

Our review used a very stringent methodology, including hand-searching of 12 relevant journals, and should have captured the most relevant RCTs. This is reflected in the full score of the AMSTAR 2, apart from one noncritical item for not including grey literature. It is still possible to miss relevant papers and we only included English-language papers. Another problem is that studies with positive findings tend to be published more often than studies with a negative finding, creating a publication bias. However, the funnel plots in our review did not show any major publication bias. Another strength of this review is that it is registered on PROSPERO, so the study protocol is publicly available (www.crd.york.ac.uk/prospero).

### Future direction

The overmedication of people with ASD is a cause for major concern, and NHS England launched a major initiative in 2016, called STOMP (STopping Over Medication of People with learning disability, autism or both), to address this issue.^49^ Therefore, it is very important to use medication for people with ASD that is based on strong evidence. Often, people with ASD and/or IDD are excluded from major intervention studies, as recruiting and obtaining informed consent is perceived as difficult. This problem can be addressed by developing appropriate ASD- and IDD-specific communication means to convey the research-related information to the participants, and using accessible easy-read versions of consent forms. It is important to include both people with ASD and their family/carers in any future research for successful recruitment. Also, the research question has to be tightly defined. For example, the current review did not find any study of antidepressants specifically for the treatment of depression or anxiety disorders. Similarly, no study was detected on the efficacy of anti-anxiety medications in treating anxiety disorders. Future research should learn from previous research as elaborated in our review, and have a multicentre, wider consensus-based approach, with very stringent scientific design.

## Data Availability

Data availability does not apply to this article as no new data were created or analysed in this study.

## Supplementary material

For supplementary material accompanying this paper visit http://dx.doi.org/10.1192/bjo.2021.1003. click here to view supplementary material
