# Supplementary material for: Randomised controlled trials of antidepressant and anti-anxiety medications for people with autism spectrum disorder: systematic review and meta-analysis
Source: BJPsych Open. 2021 Oct 1;7(6):e179. doi: 10.1192/bjo.2021.1003 (PMC8503912; doi:10.1192/bjo.2021.1003)
Supplement: Supplementary file 1 [file bjosup.zip › S2056472421010036sup001.docx]

**Appendix 1**: Search terms

Tricyclic* OR Antidepressant* OR Antidepressant drugs OR Tranquilizing drugs OR TCA OR Antianxiety OR Anxiolytics OR Anxiolytic drug* OR mood stabilizers OR Antidepressive Agents OR amitriptyline OR amitriptyline hydrochlorid OR Amoxapine OR Clomipramine or clomipramine hydrochlorid OR Dothiepin OR dosulepin hydrochloride OR dothiepin hydrochloride OR Doxepin OR Imipramine OR imipramine hydrochloride OR Lofepramine OR Nortriptyline OR Trimipramine OR Desipramine OR Florpiramine OR Dibenzepin OR Iprindole OR Protriptyline OR Maprotiline OR Opipramol OR Serotonin Uptake Inhibitors OR selective serotonin reuptake inhibitors OR SSRI OR Fluvoxamine OR Fluoxetine OR Paroxetine OR Sertraline OR Citalopram OR Escitalopram OR Venlafaxine OR Mirtazapine OR Agomelatine OR Zuclopenthixol OR Zuclopentixol OR Duloxetine OR Reboxetine OR Tryptophan OR Vortioxetine OR Benzodiazepine* OR Diazepam OR Lorazepam OR Clonazepam OR Midazolam OR Nitrazepam OR Beta-blocker* OR Propranolol OR Atenolol OR Nadolol OR Oxprenolol OR Buspirone OR Buspar OR Pregabalin OR Trazadone.

Subject Headings Used: Antidepressive Agents/Antianxiety Agents/Tranquilizing Agents (all exploded to cover subheadings)

AND

Child Development Disorder* OR pervasive developmental disorder* OR autis* OR PDD* OR ASD* OR Kanner* OR Asperger* OR Asperger* syndrome OR Autism spectrum disorder OR Rett Syndrome OR Childhood schizophrenia OR Fragile X syndrome OR Neurodevelopmental disorder* OR NDD*.

Subject Headings Used: Autistic Disorder/ Child Developmental Disorders (Pervasive)/Asperger Syndrome/Schizophrenia Childhood/Fragile X Syndrome/Pervasive Developmental Disorder (not otherwise specified) (all exploded to cover subheadings)

AND

Anxiety OR Anxiety disorder OR Depression OR Obsessive Compulsive Disorder OR OCD OR Generalised Anxiety Disorder OR Phobia OR Agoraphobia OR Autism core symptoms OR ASD core symptoms OR ASD symptoms OR Autism symptoms OR Social interaction OR Communication problems OR Behavioural problems OR Challenging behaviour OR Behaviour* that challenge OR Behaviour of concern OR Problem behaviour OR Maladaptive behaviour OR Disruptive behaviour OR Disturbed behaviour OR Distressed behaviour OR Stereotypy OR Restricted behaviour Or Repetitive patterns of behaviour OR Restricted interests OR Restrictive activities OR Social communication OR Repetitive behaviour OR Communication* OR Inattention OR Hyperactivity OR Insistence on sameness OR Sameness OR Irritability OR Sleep problem OR Insomnia OR Self injurious behaviour OR Self-mutilation OR Temper tantrum OR Tantrum OR Aggression OR Aggression to others OR Aggression to property OR Sexual aggression OR Sexual deviance OR Mental state OR Global improvement OR Quality of life OR CGI.

Subject Headings Used: Anxiety/Depression/Obsessive Compulsive Disorder/Phobic Disorders/Aggression/Social Behavior Disorders/Insomnia/Self Injurious Behavior (all exploded to cover subheadings)

AND

Clinical trial* OR Randomization* OR research design OR randomized controlled trial OR Randomi#ed control* Trial* OR RCT OR controlled clinical trial OR double-blind procedure OR Random* OR Trial* OR Control* OR Blind* OR crossover OR Crossover procedure OR Crossover trial* OR Volunteer* OR placebo* OR randomly OR control* OR ((singl* or doubl* or trebl* or tripl*) adj3 (blind* or mask*)) OR comparative stud* OR Psychopharmacology

Subject Headings: Psychopharmacolgy/Crossover Design/Clinical Trials/Comparative Studies/Randomized Controlled Trials (all exploded to cove subheadings)

NOT

Animal OR non-human OR Treatment Effectiveness Evaluation OR Treatment Outcomes OR Follow up Studies OR Evaluation*

Date Range: 1985 (January) to 2020 (October)

**Appendix 2**: Eligibility criteria

Name of the rater:

Date of rating:

Title of the paper:

Author:

Year of publication:

Name of the journal:

| Study Characteristics | Eligibility criteria  *(Insert inclusion criteria for each characteristic as defined in the Protocol)* | | Eligibility criteria met? | | |
| --- | --- | --- | --- | --- | --- |
|  |  |  | Yes | No | Unclear |
| Type of study | Randomised Controlled Trial | |  |  |  |
| Participants | Diagnosed with ASD | |  |  |  |
| Types of intervention | Antidepressants and/or anti-anxiety medication | |  |  |  |
| Types of comparison | Placebo or another medication | |  |  |  |
| Types of outcome measures | ASD core symptoms and/or other associated behavioural symptoms and/or depression and/or anxiety | |  |  |  |
| INCLUDE | | EXCLUDE UNCERTAIN | | | |
| Reason for exclusion |  | | | | |
| Notes: Get full paper if uncertain or for inclusion | | | | | |

**Appendix 3:** Excluded studies with the reason for their exclusion

| **Study author and date** | **Reason for excluding the paper** |
| --- | --- |
| Zamzow et al., 2017 | A crossover trial of propranolol vs. placebo involving 20 participants with ASD. However, propranolol was administered in a single dose. |
| Zamzow et al., 2016 | A crossover trial of propranolol vs. placebo involving 20 participants with ASD. However, propranolol was administered in a single dose. |
| Greiss Hess et al., 2016 | Children with Fragile X syndrome were included. Although summary outcome data on ASD children are presented they are not complete. |
| Chantiluke et al., 2015 Psychopharmacology | Patients were scanned twice, under either a single dose of fluoxetine or placebo in an RCT. Outcome measures were fMRI findings, rather than clinical. |
| Chantiluke et al., 2015, Psychological Medicine | Patients were scanned twice, under either a single dose of fluoxetine or placebo in an RCT. Outcome measures were fMRI findings, rather than clinical. |
| Zamzow et al., 2014 | An RCT of 14 participants with ASD and 14 matched controls participated in two study sessions in which propranolol and placebo were administered in a single dose. |
| Beversdorf et al., 2011 | An RCT of 14 participants with autism and 14 matched controls involving a single dose administration of propranolol 40 mgs vs. placebo. |
| Niederhofer et al., 2003 | A crossover trial of tianeptine vs. placebo. However, tianeptine is now withdrawn from the market because of its addictive adverse effect. |
| McDougle et al., 1996 | Investigated the behavioral and biochemical responses to acute tryptophan depletion in drug-free adults with autistic disorder. No medication was used. |

**Appendix 4:** Data extraction proforma

Notes on using data extraction form:

- Be consistent in the order and style you use to describe the information for each report.
- Record any missing information as unclear or not described, to make it clear that the information was not found in the study report(s), not that you forgot to extract it.
- Include any instructions and decision rules on the data collection form, or in an accompanying document. It is important to practice using the form and give training to any other authors using the form.

Title of the systematic review:

**General Information**

| Date form completed *(dd/mm/yyyy)* |  |
| --- | --- |
| Name/ID of person extracting data |  |
| Reference citation (full citation) |  |
| Study author contact details (Email) |  |
| Publication type *(e.g. full report, abstract, letter)* |  |
| Notes: | |

Characteristics of the included study

**Participants**

|  | Description  *Include comparative information for each intervention or comparison group if available* | |
| --- | --- | --- |
| Population description *(from which study participants are drawn)* |  | |
| Setting *(e.g. intensive care unit, service providers, institutions, day care centre etc)* |  | |
| Method of recruitment of participants *(e.g. phone, mail, clinic patients)* |  | |
| Informed consent obtained | Yes No Unclear |  |
| Intervention group | Age of participants (range, mean & SD) |  |
|  | Number (%) of participants by gender |  |
|  | Number (%) with ID, ADHD or other NDDs |  |
|  | Type of pharmacological regime (antidepressants/ antianxiety) + name of medication + dose |  |
|  | Co morbidity (psychiatric) |  |
|  | Co morbidity (physical) |  |
|  | Adverse events (number and %) |  |
| Control group | Age of participants (range, mean & SD) |  |
|  | Number (%) of participants by gender |  |
|  | Number (%) with ID, ADHD or other NDDs |  |
|  | Type of pharmacological regime (placebo or another medication) + name + dose |  |
|  | Co morbidity (psychiatric) |  |
|  | Co morbidity (physical) |  |
|  | Adverse events (number and %) |  |

**Methods**

|  | Descriptions as stated in report/paper | Location in text or source *(pg & ¶/fig/table/other)* |
| --- | --- | --- |
| Aim of study *(e.g. efficacy, equivalence, pragmatic)* |  |  |
| Design *(e.g. parallel, crossover)* |  |  |
| Sampling technique (e.g. random) |  |  |
| Method of establishing ASD diagnosis (if known) (clinical or ICD or DSM or ADI-R or ADOS etc.) |  |  |

**Outcomes**

*Copy and paste table for each outcome.*

**Outcome 1**

|  | Description as stated in report/paper | | | | | | | Location in text or source *(pg & ¶/fig/table/other)* |
| --- | --- | --- | --- | --- | --- | --- | --- | --- |
| Primary outcome if dichotomous (e.g. %) (name the outcome and the instrument used to measure the outcome) | Number (%) in the intervention arm | Total number of participants in the intervention arm | | Number (%) in the control arm | | | Total number of participants in the control arm |  |
|  |  |  | |  | | |  |  |
| Primary outcome if continuous | Mean in the intervention arm | SD in the intervention arm | Mean in the control arm | | | SD in the control arm | |  |
|  |  |  |  | | |  | |  |
| Duration of intervention (weeks/months) (if crossover, add duration of baseline and washout period) |  | | | | | | |  |
| Duration of follow up (weeks/months) |  | | | | | | |  |
| Statistical methods used and appropriateness of these *(e.g. proportion, %, risk ratio, odds ratio)* |  | | | | | | |  |
| Secondary outcomes |  | | | | | | |  |
| Number of missing data |  | | | | | | |  |
| Reason for missing data |  | | | | | | |  |
| Other |  | | | | | | |  |
| Is outcome/tool validated? | Yes No Unclear | | | | Name of the tool: | | |  |
| Notes: | | | | | | | | |

**Other information**

|  | Description as stated in report/paper | Location in text or source *(pg & ¶/fig/table/other)* |
| --- | --- | --- |
| Main findings (statistically significant difference or not; provide P value or other relevant data in support of main findings (primary and secondary outcomes) |  |  |
| Key conclusions of study authors |  |  |
| Your critique of the study (any design flaw etc.) |  |  |
| Your own overall conclusion |  |  |
| Correspondence required for further study information *(from whom, what and when)* |  | |
| Notes: | | |

**Other**

| Study funding sources *(including role of funders)* |  |  |
| --- | --- | --- |
| Possible conflicts of interest *(for study authors)* |  |  |
| Notes: | | |

**Appendix 5**: Cochrane Risk of Bias proforma

*See* [*Chapter 8*](http://www.mrc-bsu.cam.ac.uk/cochrane/handbook/index.htm#chapter_8/8_assessing_risk_of_bias_in_included_studies.htm) *of the Cochrane Handbook. Additional domains may be added for non-randomised studies.*

| Domain | Risk of bias | | | Support for judgement  *(include direct quotes where available with explanatory comments)* | Location in text or source *(pg & ¶/fig/table/other)* |
| --- | --- | --- | --- | --- | --- |
|  | Low | High | Unclear |  |  |
| Random sequence generation  *(selection bias)* |  |  |  |  |  |
| Allocation concealment  *(selection bias)* |  |  |  |  |  |
| Blinding of participants and personnel  *(performance bias)* |  |  |  | Outcome group: All/ |  |
| *(if separate judgement by outcome(s) required)* |  |  |  | Outcome group: |  |
| Blinding of outcome assessment  *(detection bias)* |  |  |  | Outcome group: All/ |  |
| *(if separate judgement by outcome(s) required)* |  |  |  | Outcome group: |  |
| Incomplete outcome data  *(attrition bias)* |  |  |  | Outcome group: All/ |  |
| *(if separate judgement by outcome(s) required)* |  |  |  | Outcome group: |  |
| Selective outcome reporting?  *(reporting bias)* |  |  |  |  |  |
| Other bias |  |  |  |  |  |
| Notes: | | | | | |

**Appendix 6:** Funnel plot CYBOCS

**
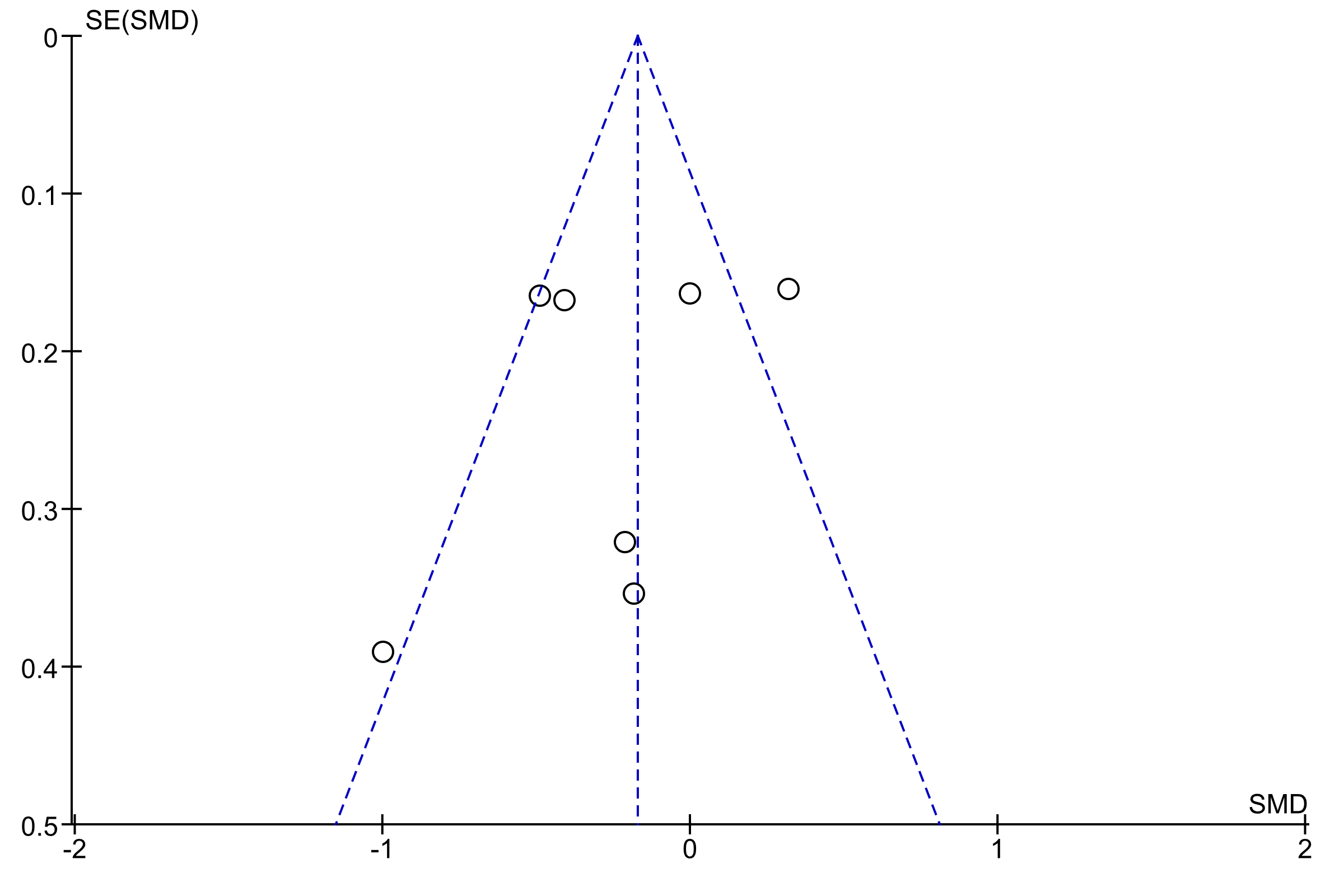
**

**Appendix 7:** Funnel plot CGI-I

**
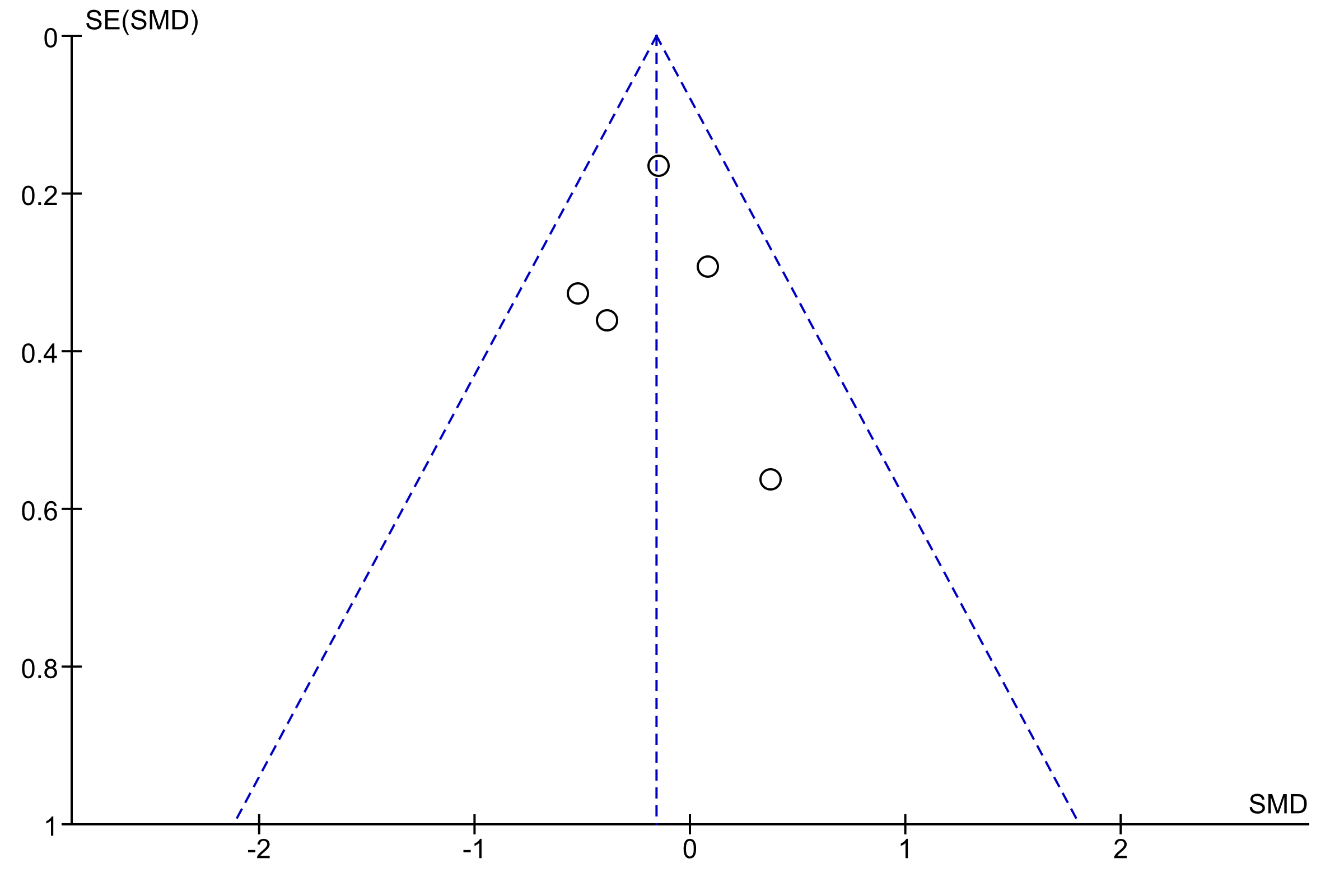
**

**Appendix 8**: AMSTAR 2 checklist

| 1. Did the research questions and inclusion criteria for the review include the components of PICO? | | | | | |
| --- | --- | --- | --- | --- | --- |
| For Yes:   - Population - Intervention - Comparator group - Outcome | | Optional (recommended)   Timeframe for follow-up | x   | Yes No |  |
| **2. Did the report of the review contain an explicit statement that the review methods were established prior to the conduct of the review and did the report justify any significant deviations from the protocol?** | | | | | |
|  | For Partial Yes:  The authors state that they had a written protocol or guide that included ALL the following:   - review question(s) - a search strategy - inclusion/exclusion criteria - a risk of bias assessment | For Yes:  As for partial yes, plus the protocol should be registered and should also have specified:   - a meta-analysis/synthesis plan, if appropriate, *and* - a plan for investigating causes of heterogeneity - justification for any deviations from the protocol | x     | Yes Partial Yes No |  |
| **3. Did the review authors explain their selection of the study designs for inclusion in the review?** | | | | | |
|  | For Yes, the review should satisfy ONE of the following:   - *Explanation for* including only RCTs - OR *Explanation for* including only NRSI - OR *Explanation for* including both RCTs and NRSI | |  | Yes |  |
| **4. Did the review authors use a comprehensive literature search strategy?** | | | | | |
|  | For Partial Yes (all the following): | For Yes, should also have (all the following):   - searched the reference lists / bibliographies of included studies - searched trial/study registries - included/consulted content experts in the field - where relevant, searched for grey literature - conducted search within 24 months of completion of the review |  |  |  |
|  | - searched at least 2 databases (relevant to research question) - provided key word and/or search strategy - justified publication restrictions |  |   x   | Yes Partial Yes No |  |
|  | (e.g. language) |  |  |  |  |
|  | **5. Did the review authors perform study selection in duplicate?** | |  | |  |
|  | For Yes, either ONE of the following:   - at least two reviewers independently agreed on selection of eligible studies and achieved consensus on which studies to include - OR two reviewers selected a sample of eligible studies and achieved good agreement (at least 80 percent), with the remainder selected by one reviewer. | | x   | Yes No |  |

| **6. Did the review authors perform data extraction in duplicate?** | | | | | | | |
| --- | --- | --- | --- | --- | --- | --- | --- |
| For Yes, either ONE of the following:   - at least two reviewers achieved consensus on which data to extract from included studies - OR two reviewers extracted data from a sample of eligible studies and achieved good agreement (at least 80 percent), with the remainder extracted by one reviewer. | | | | | | x Yes   - No | |
| **7. Did the review authors provide a list of excluded studies and justify the exclusions?** | | | | | | | |
|  | | For Partial Yes:   provided a list of all potentially relevant studies that were read  in full-text form but excluded from the review | For Yes, must also have:   Justified the exclusion from the review of each potentially relevant study | | | x Yes   - Partial Yes - No | |
| **8. Did the review authors describe the included studies in adequate detail?** | | | | | | | |
|  | | For Partial Yes (ALL the following):   - described populations - described interventions - described comparators - described outcomes - described research designs | For Yes, should also have ALL the following:   - described population in detail - described intervention in detail (including doses where relevant) - described comparator in detail (including doses where relevant) - described study’s setting - timeframe for follow-up | | | x Yes   - Partial Yes - No | |
| **9. Did the review authors use a satisfactory technique for assessing the risk of bias (RoB) in individual studies that were included in the review?** | | | | | | | |
|  | | **RCTs**  For Partial Yes, must have assessed RoB from   - unconcealed allocation, *and* - lack of blinding of patients and assessors when assessing outcomes (unnecessary for objective outcomes such as all-   cause mortality) | For Yes, must also have assessed RoB from:   - allocation sequence that was not truly random, *and* - selection of the reported result from among multiple measurements or analyses of a specified outcome | | | x Yes   - Partial Yes - No - Includes only NRSI | |
|  | | **NRSI**  For Partial Yes, must have assessed RoB:   - from confounding, *and* - from selection bias   **10. Did the review authors report o** | For Yes, must also have assessed RoB:   - methods used to ascertain exposures and outcomes, *and* - selection of the reported result from among multiple measurements or analyses of a specified outcome   **n the sources of funding for the studies inc** | | | - Yes - Partial Yes - No - Includes only RCTs   **luded in the review?** | |
|  | | For Yes   Must have reported on the sources of funding for individual studies included x Yes in the review. Note: Reporting that the reviewers looked for this information  No but it was not reported by study authors also qualifies | | | | | |
| **11. If meta-analysis was performed did the review authors use appropriate methods for statistical combination of results?** | | | | | | | |
|  | **RCTs**  For Yes:   - The authors justified combining the data in a meta-analysis   - AND they used an appropriate weighted technique to combine study results and adjusted for heterogeneity if present.   - AND investigated the causes of any heterogeneity | | | | x Yes   - No - No meta-analysis conducted | |  |
|  | **For NRSI**  For Yes:   - The authors justified combining the data in a meta-analysis   - AND they used an appropriate weighted technique to combine study results, adjusting for heterogeneity if present   - AND they statistically combined effect estimates from NRSI that were adjusted for confounding, rather than combining raw data, or justified combining raw data when adjusted effect estimates were not available   - AND they reported separate summary estimates for RCTs and NRSI separately when both were included in the review | | | | - Yes - No - No meta-analysis conducted | |  |
| **12. If meta-analysis was performed, did the review authors assess the potential impact of RoB in individual studies on the results of the meta-analysis or other evidence synthesis?** | | | | | | | |
|  | For Yes:   - included only low risk of bias RCTs - OR, if the pooled estimate was based on RCTs and/or NRSI at variable RoB, the authors performed analyses to investigate possible impact of RoB on summary estimates of effect. | | | | x Yes   - No - No meta-analysis conducted | |  |
| **13. Did the review authors account for RoB in individual studies when interpreting/ discussing the results of the review?** | | | | | | | |
|  | For Yes:   - included only low risk of bias RCTs - OR, if RCTs with moderate or high RoB, or NRSI were included the review provided a discussion of the likely impact of RoB on the results | | | | x Yes   - No | |  |
| **14. Did the review authors provide a satisfactory explanation for, and discussion of, any heterogeneity observed in the results of the review?** | | | | | | | |
|  | For Yes:   - There was no significant heterogeneity in the results - OR if heterogeneity was present the authors performed an investigation of sources of any heterogeneity in the results and discussed the impact of this on the results of the review | | | | x Yes   - No | |  |
| **15. If they performed quantitative synthesis did the review authors carry out an adequate investigation of publication bias (small study bias) and discuss its likely impact on the results of the review?** | | | | | | | |
|  | For Yes:   performed graphical or statistical tests for publication bias and discussed the likelihood and magnitude of impact of publication bias | | | | x Yes   - No - No meta-analysis conducted | |  |
| **16. Did the review authors report any potential sources of conflict of interest, including any funding they received for conducting the review?** | | | | | | | |
|  | For Yes:   - The authors reported no competing interests OR - The authors described their funding sources and how they managed potential conflicts of interest | | | x Yes   - No | | |  |

**Appendix 9**: Inclusion, exclusion criteria, comorbidities and study funding source

| Study | Participant inclusion criteria | Comorbidity (psychiatric) | Comorbidity (physical) | Source of funding |
| --- | --- | --- | --- | --- |
| **Agomelatine** | | | | |
| Ballester et al. (2019) | Inclusion criteria:  Age between 18 and 45 years. ASD According to DSM-5 criteria and two clinicians. Agreement to follow up visits. Sleep problems according to ICSD3.  Exclusion criteria:  Intake of CYP 1A2 inhibitors | Mood disorders (58%)  Aggressive Behaviours (12%)  Anxiety (5%) | CNS conditions (87%) | Alicia Koplowitz Charitable Foundation (UGP-14-011), Servier Laboratories SL |
| **Buspirone** | | | | |
| Chugani et al. (2016) | Inclusion criteria:  2-6 years (grouped as 2-4 and 4-6 years). ASD diagnosis based on DSM-IV criteria, ADI-R and ADOS | Not specified | Not specified | National Institute of Neurological Disorders and Stroke (cooperative agreement 5U01 NS61264) |
| Ghanizadeh et al. (2015) | Inclusion criteria:  Mean age 7.05 years. Outpatients with autism diagnosis | Not specified | Not specified | Shiraz University of Medical Sciences (grant no. 6978) |
| **Citalopram** | | | | |
| King et al. (2009) | Inclusion criteria:  Age range 5-17 years. DSM-IV-TR criteria for autistic disorder, Asperger disorder or PDD-NOS as determined by experienced clinician using ADI-R and ADOS,  Illness severity rating of at least moderate (CGI-Severity of Illness Scale). Score at least moderate on compulsive behaviours (CYBOCS-PDD).  Exclusion criteria:  Rett disorder or childhood disintegrative disorder, a seizure within the past 6 months, weight less than 15 kg, a medical condition that might interfere with study participation, clinically significant abnormal  baseline laboratory test results, history of adverse events or failed treatment while taking two or more SSRIs, prior treatment with citalopram or escitalopram oxalate, recent initiation of behavioural therapy, or history of bipolar disorder or manic episode | Not specified (see exclusion criteria) | Not specified (see exclusion criteria) | National Institutes of Health: Mount Sinai School of Medicine, New York: (U54-MH066673), University of North Carolina at Chapel Hill: (U54-MH066418), University of California at Los Angeles: (U54-MH068172), Yale University, New Haven, Connecticut: (U54-MH066494), Dartmouth Medical School, Hanover, New Hampshire, and Boston University, Boston, Massachusetts: (U54-  MH066398), Boston: (U01-HD045023). The study was monitored by National Institute of Mental Health Data and Safety Management Board A. |
| **Clomipramine** | | | | |
| Gordon et al. (1993) | Inclusion criteria:  Mean age 11.7 years. Diagnosis of ID.  Exclusion criteria:  Physical medical problems | Not specified (see exclusion criteria) | Not specified (see exclusion criteria) | Not specified |
| Remington et al. (2001) | Inclusion criteria:  Age range 10-36 years, mean age 16.3 years. DSM-IV diagnosis of autism confirmed independently by two of the investigators both of whom specialize in autistic disorder | Not specified | Not specified | Ontario Mental Health Foundation |
| **Fluoxetine** | | | | |
| Herscu et al. (2020) | Inclusion criteria:  Age range 5-17 years. Autistic disorder diagnosis based on all three of DSM-IV-TR, Autism Diagnostic Interview-Revised and Autism Diagnostic Observation Schedule-Generic  CYBOCS-PDD score >=10.  Exclusion criteria:  Asperger’s Disorder, PDD-NOS, Rett Syndrome, Childhood Disintegrative Disorder. Active seizure disorder, current or previous use of fluoxetine, current use of any other psychotropic medication, recent initiation of behaviour therapy, or concomitant use of any of the contraindicated medications listed on the fluoxetine package insert. Individuals with high levels of aggression, irritability, or self-injurious behaviour that would be more appropriately treated with a psychotropic medication other than an SSRIs.  Clinically significant abnormal baseline laboratory test results or medical condition that might interfere with study participation | Not specified (see exclusion criteria) | Not specified (see exclusion criteria) | Neuropharm Plc. in collaboration with the Autism Speaks Autism Clinical Trials Network |
| Hollander et al. (2005) | Inclusion criteria:  Age range 5-17 years. ASD criteria-including autism, Asperger Syndrome, Pervasive Developmental Disorder not otherwise specified as per ADI-R, ADOS-G or DSM-IV-TR by psychiatric interview including information about timing and quality of early language.  Exclusion criteria:  DSM-IV psychotic disorders, history of seizures or clinically significant medical illness | Not specified (see exclusion criteria) | Not specified (see exclusion criteria) | Orphan Products Division of the Food and Drug Administration Grant # FD-R-001520-01-03, NIH STAART Center of Excellence Grant #1U54 MM066673-01A1, NARSAD Young Investigator Award for Dr Novotny, and the Seaver Foundation. Lilly Research Laboratories provided liquid fluoxetine and matching placebo for the study. |
| Reddihough et al. (2019) | Inclusion criteria:  Age range 7.5 to 18 years. ASD diagnosis according to ADI-R and DSM-IV-TR, including autistic disorder, Asperger disorder and PDD-NOS.  Exclusion criteria:  Children and adolescents with a diagnosis of Rett syndrome, childhood disintegrative disorder, schizophrenia, or major depression, had previously received fluoxetine, or were currently prescribed or had received in the 6-week period before study entry other SSRIs,  psychotropic medications (including typical and atypical antipsychotics, mood stabilizers, and anxiolytics), monoamine oxidase inhibitors or pimozide, antidepressants, or St John’s wort. Children with significant comorbid medical conditions and also epilepsy | Not specified (see exclusion criteria) | Not specified (see exclusion criteria) | NHMRC (NHMRC project grant 607332). Infrastructure support was provided by the Victorian government’s Operational Infrastructure Support Program. The Murdoch Children’s Research Institute, Melbourne, was the sponsor for this study. Role of the Funder/Sponsor: The agencies providing funding/support for this work had no role in the design and conduct of the study; collection, management, analysis, and interpretation of the data; preparation, review, or approval of the manuscript; and decision to submit the manuscript for publication |
| Hollander et al. (2012) | Inclusion criteria:  Age range 18-60 years. ASD diagnosis according to DSM-IV criteria, through clinic evaluation by psychiatrists and neurologists with substantial experience with adult ASDs, via ADOS-Generic and ADI-Revised. Some who met only the social interaction criteria of ADOS, but ADI results were consistent with DSM-IV diagnosis were included.  Exclusion criteria:  Schizophrenia, schizoaffective disorder, bipolar disorder, active seizure disorder, significant hematopoietic or cardiovascular disease | Not specified (see exclusion criteria) | Not specified (see exclusion criteria) | Food and Drug Administration orphan product grant FD-R-002026-01 and supported by Studies to Advance Autism Re-search and Treatment (STA A RT) Center of Excellence grant 1U54MH-066673 from NIMH, by the Seaver Foundation, and by the Mount Sinai General Clinical Research Center. Mount Sinai School of Medicine licensed an orphan designation for fluoxetine in autism to Neuropharm, Ltd. |
| **Fluvoxamine** | | | | |
| McDougle et al. (1996) | Inclusion criteria:  Adults diagnosed with autism based on DSM III-R and ICD10 Revision criteria for autistic disorder using ADI and ADOS by trained professionals.  Exclusion criteria:  Schizophrenia or had psychotic symptoms, if had abused illicit substances in previous six months. Notable medical conditions, seizure disorder, women with positive serum pregnancy test results | Not specified (see exclusion criteria) | Not specified (see exclusion criteria) | National Alliance for Research on Schizophrenia and Depression Young Investigator Award (Dr McDougle), The Korczak Foundation for Autism and Related Disorders, M01 RR06022-33, P50 MH30929-18, HD 03008-27, and P01 MH25642 from the National Institutes of Health, Bethesda, Md. Fluvoxamine and financial support were provided by Solvay Pharmaceuticals, Marietta, Ga |
| Sugie et al. (2005) | Inclusion criteria:  Japanese children with autistic disorder diagnosed by paediatric neurologists and clinical psychologists based on DSM-IV.  Exclusion criteria:  Patients with evident underlying diseases, such as chromosomal aberration, congenital rubella syndrome and apparent neurological deficits, on any psychotropic drugs for at least four weeks before the trial | Not specified (see exclusion criteria) | Not specified (see exclusion criteria) | Not specified |
| **Sertraline** | | | | |
| Potter et al. (2009) | Inclusion criteria:  Age range 24-72 months. ASD diagnosis according to DSM-V and ADOS-2. Stable medications (including antiepileptics, antipsychotics, and clonidine) in the two months prior to enrolment. Concurrent enrolment in at least one community or school intervention for ASD.  Exclusion criteria:  Current or past SSRI treatment, diagnosis of the Fragile X syndrome full mutation, or any other serious co-morbid medical disorders affecting brain function and behaviour, including uncontrolled seizures | Not specified (see exclusion criteria) | Not specified (see exclusion criteria) | Health Resources and Services Administration (HRSA) of the U.S. Department of Health and Human Services (HHS) under grant number R40MCH 27701. MIND Institute Intellectual and Developmental Disabilities Research Center which is funded by the National Institute of Child Health and Human Development (U54 HD079125). National Center for Advancing Translational Sciences and National Institutes of Health (grant UL1 TR001860). |
| **Venlafaxine** | | | | |
| Carminati et al. (2016) | Inclusion criteria:  Age between 18 and 30 years. Diagnosis of intellectual disability associated with ASD. ICD-10 diagnosis of intellectual disability, mild to profound with pervasive development disorders.  Exclusion criteria:  Epilepsy or indication against psychotropic treatments, pregnancy | Not specified (see exclusion criteria) | Not specified (see exclusion criteria) | Fondation Handicap Mental & Société (FHMS), Geneva, Switzerland, (N. CGR 73166 PS-Venlafaxine). The funding sources had no role in study design; in the collection, analysis and interpretation of data; in the writing of the report; and in the decision to submit the article for publication |
| Niederhof et al. (2004) | Inclusion criteria:  Age range 5.2-11.7 years. Outpatient male children. ICD-10 criteria for autistic disorder, with agreement of the independent diagnosis of autistic disorder by at least two child and adolescent psychiatrists. Social and pragmatic language deficits consistent with autistic disorder. Off medication for at least one month before the study onset.  Exclusion criteria:  History of identified medical or neurologic illnesses | Not specified (see exclusion criteria) | Not specified (see exclusion criteria) | Not specified |

ADI: Autism Diagnostic Interview; ADI-R: Autism Diagnostic Interview-Revised; ADOS: Autism Diagnostic Observation Schedule; ADOS-2: Autism Diagnostic Observation Schedule, 2nd Edition; ADOS-G: Autism Diagnostic Observation Schedule-Generic; ASD: Autism Spectrum Disorder; CGI-S: Clinical Global Impressions-Severity Scale; CNS: Central Nervous System; CYBOCS-PDD: Children’s Yale-Brown Obsessive Compulsive Scale-Modified for Pervasive Developmental Disorders; CYP1A2: Cytochrome P450 1A2; DSM III-R: Diagnostic and Statistical Manual of Mental Disorders, 3rd Edition, revised; DSM-5/DSM-V: Diagnostic and Statistical Manual of Mental Disorders, 5th Edition; DSM-IV: Diagnostic and Statistical Manual of Mental Disorders, 4th Edition; DSM-IV-TR: Diagnostic and Statistical Manual of Mental Disorders, 4th Edition Text Revision; ICD-10: International Classification of Diseases 10th Revision; ICSD3: The International Classification of Sleep Disorders, 3rd Edition; ID: Intellectual Disabilities; Kg: Kilogram; PDD-NOS: Pervasive Developmental Disorder-Not Otherwise Specified; SSRI: Selective Serotonin Reuptake Inhibitor.
