# Supplementary material for: Randomised controlled trials of antidepressant and anti-anxiety medications for people with autism spectrum disorder: systematic review and meta-analysis
Source: BJPsych Open. 2021 Oct 1;7(6):e179. doi: 10.1192/bjo.2021.1003 (PMC8503912; doi:10.1192/bjo.2021.1003)
Supplement: Supplementary file 1 [file bjosup.zip › S2056472421010036sup002.docx]

Table 1 Summary findings

| Study author and date | Study type, (methods used for ASD diagnosis) | Dose of medications | Participants (N, Age (mean ± s.d.), gender, IQ (mean ± s.d.), Intervention, FU | Outcome measures used | Findings, Jadad score | Comments |
| --- | --- | --- | --- | --- | --- | --- |
| Agomelatine | | | | | | |
| Ballester et al, 2019^20^ | Crossover (DSM-V) | Agomelatine (25 mg/day) | N: 23 Age: 18–65 years (53 ± 12 years) Male: 83% IQ: 100% IDD Agomelatine/Placebo: 23 FU: 1 month | TST, TiB, SoL, Number of awakenings during TiB, Wake after sleep onset, Sleep efficiency | Only night TST significantly increased (mean 83 min) during agomelatine treatment (abnormal TST among 50% participants pre-treatment *v*. 16% post agomelatine treatment) (*P* = 0.016). No significant change in other sleep parameters The authors reported only mild and transient adverse events associated with the agomelatine treatment. Jadad = 4 | Small sample size risking type II error. The outcome is not directly related to ASD core symptoms, although sleep problem is common in ASD. Only one sleep parameter has shown improvement. |
| Buspirone | | | | | |  |
| Chugani et al, 2016^21^ | Parallel design (DSM-IV, ADI-R and ADOS) | Buspirone 2.5 mg twice daily *v*. Buspirone 5 mg twice daily | *N* = 166 (142 completed) Age: 2–6 years Male: 82.5% IQ: Not reported Buspirone 2.5 mg twice daily = 54 Buspirone 5 mg twice daily = 55 Placebo = 57 FU: 24 weeks | ADOS-CTS, ADOS-SA and ADOS-RRB, ABC, VABS, RBS, SPS, C-YBOCS-PDD, CGI, Leiter Parent-Report | A non-significant intergroup difference in the primary outcome ADOS-CTS. RRB score as per CYBOCS-PDD showed a significant difference from baseline to FU (*P* = 0.03) in 2.5 mg twice daily dose but not in the 5 mg twice daily dose or the placebo group There was no significant intergroup difference in the rate of adverse events. Jadad = 5 | Reasonable sample size but contradictory findings based on different dosages and outcome measures. |
| Ghanizadeh & Ayoobzadehshirazi, 2015^22^ | Parallel design (Clinical diagnosis) | Buspirone 10 mg/day (<40 kg) or 20 mg/day (>40 kg) | N: 40 (34 completed) Age: Mean: 7 years Male: 82.5% IQ: Not reported Buspirone: 16 Placebo: 18 FU: 4 and 8 weeks | ABC-Irritability subscale | Thirteen (81.2%) in the buspirone group and seven (38.9%) in the placebo group showed a >30% decline in irritability score, although total irritability score improved significantly from the baseline score in both groups (both *P* < 0.001). No major adverse events are reported. The most common adverse events associated with buspirone treatment were increased appetite (61%), drowsiness (11%), and fatigue (11%) Jadad = 5 | Short study duration. Small sample size risking a type II error. |
| Citalopram | | | | | | |
| King et al, 2009^23^ | Parallel design (DSM-IV-TR, ADI-R, ADOS) | Citalopram 10 mg/5 mL, 2.5 mg-20 mg/day | N: 149 (123 completed) Age: 5–17 years (9.4 ± 3.1 years) Male: 86% IQ: 40% had a non-verbal IQ <70 Citalopram: 60 Placebo: 63 FU: 12 weeks | C-YBOCS-PDD total score + RRB, CGI-I, RBS, ABC | No significant intergroup difference in C-YBOCS-PDD, RRB, and CGI-I scores Citalopram group showed more adverse effects such as increased energy, impulsiveness, decreased concentration, hyperactivity, stereotypy, diarrhoea, insomnia, and dry skin or pruritus. Jadad = 5 | A large sample is likely to have given adequate power to the study but the overall dropout rate maybe a bit high. However, an ITT analysis should have mitigated this. |
| Clomipramine | | | | | | |
| Gordon et al, 1993^24^ | Crossover (DSM-III-R, ADI) | Clomipramine *v*. desipramine 25 mg/day-250 mg/day (or 5 mg/kg/day) | N: 28 (24 completed) Age: 6–23 years (10.4 ± 4.1 years) Male: 62.5% IQ: 30–107 (*n* = 19; 79.16% ≤70) Clomipramine/Placebo: 12 Clomipramine/Desipramine: 12 FU: week 5 | Modified CPRS OCD subscale, CGI | Clomipramine was superior to both placebo (*P* ≤ 0.001) and desipramine (*P* ≤ 0.005) in improving CPRS subscales and CGI scores There was no effect of age, gender, IQ level, and dose of clomipramine on the outcome. The authors reported minor adverse effects in all three groups without any significant intergroup difference. Jadad = 3 | Small sample size risking a type II error. A short washout period may have caused a carry-over effect on long-standing behaviours. |
| Remington et al, 2001^25^ | Crossover Latin square (DSM-IV) | Clomipramine (100–150 mg/day, mean: 128.4 mg/day); Haloperidol (1–1.5 mg/day; mean: 1.3 mg/day) | N: 36 Age: 10–36 years, (mean: 16.3) Male: 86% IQ: Not stated Clomipramine/ Haloperidol/ Placebo: 36 FU: 7 weeks | Global ASD measures CARS, ESRS, DOTES, ABC- subscales | Clomipramine did not show any superiority over placebo, but haloperidol did (*P* < 0.05) on the global measure of autistic symptoms, and ABC-irritability, hyperactivity, and stereotype subscale scores. Adverse events associated with clomipramine included fatigue (*n*= 4), tremors (*n*= 2), tachycardia (*n*= 1), insomnia (*n*= 1), diaphoresis (*n*= 1), nausea or vomiting (*n*= 1), and decreased appetite (*n*= 1). Four of them also showed problem behaviour. Jadad = 4 | Small sample size risking a type II error. A short washout period may have caused a carry-over effect on long-standing behaviours. A complicated study design has made the interpretation of findings difficult. |
| Fluoxetine | | | | | | |
| Herscu et al, 2020^26^ (SOFIA study) | Parallel design (DSM-IV-TR) | Fluoxetine 2–18 mg/day (Mean: 11.8 mg/day) | N: 158 (121 completed) Age: 5–17 years Male: 85.5% IQ: Not reported Fluoxetine: 56 Placebo: 65 FU: 14 weeks | CYBOCS-PDD total + RRB score, CGI, CSQ | No significant intergroup difference in any of the outcome measures. ‘Much improved’ and ‘very much improved’ in 23% of fluoxetine and 34% placebo group as per CGI-I scores. The rate of adverse effects was similar in the two groups and both groups showed a high rate of activation (fluoxetine: 42%, placebo: 45%). Jadad = 5 | A large sample may have given adequate power, but the authors suggested that a low starting dose may have prevented a therapeutic effect. |
| Hollander et al, 2005^27^ | Crossover (DSM-IV, ADI-R, ADOS) | Fluoxetine 2.5 mg/day to 0.8 mg/kg/day | N: 45 (39 completed) Age: 5–17 years Male: 76.9% IQ: 30–132 (63.65 ± 27.9) Fluoxetine/Placebo: 39 FU: 20 weeks | C-YBOCS RRB score, Autism global symptoms, CGI | Moderate to large effect size on C-YBOCS RRB score (z = −2.075, SE = 0.407, *P* = 0.038). No effect on CGI, speech, social interaction. There was no significant intergroup difference in the rate of adverse effects. The fluoxetine group reported a numerically lower rate of insomnia, anxiety, urinary incontinence, and mild weight gain but a higher rate of sedation, agitation, diarrhoea, and anorexia when compared with the placebo group. Jadad = 3 | Small sample size risking a type II error. A short washout period may have caused a carry-over effect on long-standing behaviours. |
| Hollander et al, 2012^28^ | Parallel design (DSM-IV, ADI-R, ADOS) | Fluoxetine 10 mg/day starting dose to maximum of 80 mg/day | N: 37 (34 completed) Age: 18–60 years (34.31 ± 14.26 years) Male: 69% IQ: 30–161 (103.25 ± 28.45) Fluoxetine: 21 Placebo: 13 FU: 12 weeks | YBOCS RRB score, CGI, ABC, HAM-D | Fluoxetine was superior to placebo on YBOCS RRB score (F = 9.24, df = 1, 30.7, *P* = 0.005, d = 0.53). CGI improvement in obsessive-compulsive symptoms (score 2 or less): 50% in the fluoxetine group *v*. 8% in the placebo group (*P* = 0.03). HAM-D scores were well under the threshold for a diagnosis of depressive disorder in both groups. A statistical comparison of intergroup adverse effects was not possible. In the fluoxetine group, 1.4 adverse effects per patient were reported compared with 0.6 in the placebo group. Adverse events associated with the fluoxetine treatment were mild to moderate and included bad dreams (*n*= 3), mild insomnia (*n*= 3), mild dry mouth (*n*= 3), and headaches (*n*= 3). There was no statistically significant intergroup difference in the rates of suicidal ideation between the fluoxetine (6%) and the placebo group (0%) (*P* = 1.00). No one in either group reported suicidal gestures or attempts. Jadad = **3** | Small sample size risking a type II error. |
| Reddihough et al, 2019^29^ | Multicentre, parallel design (DSM-IV-TR, ADI-R) | Fluoxetine: commencing at 4 or 8 mg/day for the first week (4 mg if <40 kg; 8 mg if ≥40 kg) maximum dose was 20 mg/day (participants <40 kg) or 30 mg/day (participants ≥40 kg). | N: 146 (109 completed) Age: 7.5–18 years (11.2 ± 2.9 years) Male: 80% IQ: 30% IDD Fluoxetine: 54 Placebo: 55 FU: 16 weeks | C-YBOCS-PDD, RBS, ABC, CGI | The mean C-YBOCS-PDD score decreased in the fluoxetine group by 3.72 points (95% CI, −4.85 to −2.60) and in the placebo group by 2.53 points (95% CI, −3.86 to −1.19). The between-group mean difference at follow up was −2.01 (95% CI, −3.77 to −0.25; *P* = 0.03) (adjusted for stratification factors), and in the prespecified model with further adjustment, it was −1.17 (95% CI, −3.01 to 0.67; *P* = 0.21). The multiple imputation analysis did not show any statistically significant intergroup difference (mean difference, −1.82; 95%CI, −3.71 to 0.06; *P* = 0.06). The rate of commonly observed adverse effects such as irritability, mood disturbance, nausea, vomiting, and sleep disorders did not show any significant intergroup difference (fluoxetine, 45%, the total number of events 2.5(s.d. ± 1.6) and placebo, 48%, the total number of events 2.6(s.d. ± 2.1). Jadad = 4 | Initial analysis showed the superiority of fluoxetine over placebo, but this difference was not maintained when baseline imbalance in outcome scores between two arms was adjusted for. Also, the confidence interval of the scores included a minimally clinically important difference. High dropout rate (25%) |
| Fluvoxamine | | | | | | |
| McDougle et al, 1996^30^ | Parallel design (DSM-III-R, ICD-10) | Fluvoxamine: 50 mg/day, increased every 3 or 4 days to a maximum of 300 mg/day as tolerated | N: 30 (all completed) Age: 18–53 years (30.1 ± 7.7 years) Male: 90% IQ: 25–114 (79.9 ± 29.7) Fluvoxamine: 15 Placebo:15 FU: 12 weeks | YBOCS, CGI, VABS maladaptive behavior subscale, Brown Aggression Scale, Ritvo-Freeman Real-Life Rating Scale | A significantly higher proportion of the fluvoxamine group (53%) was reported to be responders to treatment compared with the placebo group (0%). Fluvoxamine was superior in improving repetitive thoughts and behaviour (*P* < 0.001), problem behaviour (*P* < 0.001), aggression (*P* < 0.03), some aspects of social relatedness (*P* < 0.04), and language usage (*P* < 0.008). Age, the severity of autism symptoms, and the level of IQ did not influence the treatment response. Other than mild sedation and nausea in a few cases, fluvoxamine was well tolerated. No one reported dyskinesias, seizures, electrocardiograph change, anticholinergic, or adverse cardiovascular events. Jadad = 3 | Small sample size risking a type II error. |
| Sugie et al, 2005^31^ | A crossover study in the context of genetic evaluation (DSM-IV) | Fluvoxamine 1–3 mg/kg body weight/day | N: 19 (18 completed) Age: 3 years - 8 years 5 months (mean: 5 years 4 months) Male: 79% IQ: Not reported Fluvoxamine/Placebo: 18 FU: 12 weeks | BAS, CGI | Fluvoxamine showed a statistically significant improvement in two (flighty eye movements and delayed or peculiar or inappropriate speech) of the 20 BAS items compared with the placebo. Fluvoxamine did not show any significant adverse effects other than transient nausea and hyperactivity. Jadad = 5 | Small sample size risking a type II error. No adjustment is made for multiple comparisons to avoid any type I error. |
| Sertraline | | | | | | |
| Potter et al, 2019^32^ | Parallel design (DSM-IV, ADOS) | Sertraline (20 mg/mL), under four years: 2.5 mg/day (0.125 mL); four years or older: 5 mg/day (0.25 mL). | N: 58 (45 completed) Age: 2–6 years Male: 79.5% IQ: (DQ: sertraline: 48.72 ± 28.46; placebo: 50.63 ± 24.05) Sertraline: 24 Placebo: 21 FU: 6 months | MSEL (expressive language raw score and age equivalent combined score), CGI, PVET, VABS-II, ABC-C, PAS-R, SRS, SPM-P, PLS-5, Visual Analog Scale | ITT analysis did not show any significant intergroup difference in any outcome measure. No statistically significant intergroup difference in the rate of adverse effects. No sertraline-associated serious adverse event was reported. Jadad = 4 | Small sample size risking a type II error. |
| Venlafaxine | | | | | | |
| Carminati et al, 2016^33^ | Parallel design (ICD-10) | Venlafaxine 18.75 mg/day Zuclopenthixol or clonazepam introduced or continued with dose adjustment as necessary | N: 13 Age : venlafaxine : 18–30 years (median : 22 years); placebo: 19–32 years (median : 19 years) Male: 84.5% IQ: 100% IDD Venlafaxine: 6 Placebo: 7 FU: 8 weeks | ABC, BPI, CGI Reduction in usual doses of zuclopenthixol or clonazepam, Simpson-Angus Rating Scale | Univariate analyses showed that the symptom of irritability improved in the entire sample (*P* = 0.061), although no difference was observed between the venlafaxine and the placebo group. No significant decrease in hyperactivity/noncompliance was observed during the study. Global improvement was observed in 33% of participants treated with venlafaxine and in 71% of participants in the placebo group (*P* = 0.29). Decreased cumulative doses of clonazepam and zuclopenthixol were required for the venlafaxine group. Multivariate analyses (principal component analyses) with at least three combinations of variables showed that the two populations could be clearly separated (*P* < 0.05). A few adverse effects in the venlafaxine group included excessive salivation, slight elbow stiffness, mild finger tremor, and head dropping. No comparative data for the placebo group is provided by the authors. Jadad = 3 | Small sample size. Very complicated design involving the use of additional medications, which makes interpretation of the findings difficult. Different results are shown depending on the statistical analysis used (univariate *v*. multivariate). |
| Niederhofer, 2004^34^ | Crossover (ICD-10) | Venlafaxine: 30 mg/day | N: 15 (14 completed) Age: 5.2–11.7 years (7.1 ± 3 years) Male: 100% IQ: 55-79 (67 ± 12) Venlafaxine/Placebo: 14 FU: 6 weeks | CGAS, CGI, ABC, Modified CPRS | No statistically significant intergroup difference in the clinician’s rating of videotaped behaviour according to CPRS, CGAS, and CGI. Statistically significant better improvement in ABC sub scores of irritability (*P* = 0.04), inadequate eye contact (*P* = 0.042), hyperactivity (*P* = 0.035), and lethargy (*P* = 0.043) according to the teacher’s rating during the venlafaxine treatment compared with the placebo phase. Adverse effects such as increased thirst, drowsiness, sleep disturbance, sadness, dizziness, irritability, decreased activity have been reported in the venlafaxine treatment group but without providing the number of participants affected or any comparative data in the placebo phase. Jadad = 3 | Very small all-male clinic sample, so the generalisability of the findings is questionable. |

ABC, Aberrant Behaviour Checklist; ABC-C, Aberrant Behavior Checklist-Community version; ADI-R, Autism Diagnostic Interview-Revised; ADOS-CTS, Autism Diagnostic Observation Schedule-Composite Total Score; ADOS-SA, Autism Diagnostic Observation Schedule-Social Affect; ADOS-RRB, Autism Diagnostic Observation Schedule-Restricted and Repetitive Behaviour; ASD, Autism Spectrum Disorder; BAS, Behaviour Assessment Scale; BPI, Behaviour Problems Inventory; CARS, Childhood Autism Rating Scale; CGAS, Children’s Global Assessment Scale; CGI, Clinical Global Impressions Scale; CGI-I, Clinical Global Impressions-Improvement Scale; CI, Confidence Interval, CPRS OCD subscale, Comprehensive Psychological Rating Scale Obsessive Behaviour Disorder subscale; CSQ, Caregiver Strain Questionnaire; C-YBOCS-PDD, Children’s Yale-Brown Obsessive Compulsive Scale-Modified for Pervasive Developmental Disorders; df, degree of freedom; DOTES, Dosage Treatment Emergent Symptom Scale; DSM-V, Diagnostic and Statistical Manual of Mental Disorders, 5th Edition; DSM-IV, Diagnostic and Statistical Manual of Mental Disorders, 4th Edition; DSM-IV-TR, Diagnostic and Statistical Manual of Mental Disorders, 4th Edition Text Revision; DSM-III-R, Diagnostic and Statistical Manual of Mental Disorders, 3rd Edition-Revised; DQ, Development Quotient; ESRS, Extrapyramidal Symptom Rating Scale; FU, Follow-up; ICD-10, International Classification of Diseases 10th Revision; HAM-D, Hamilton Depression Rating Scale; IDD, Intellectual and Developmental Disability; ITT, Intention to Treat; IQ, Intelligence Quotient; MSEL, Mullen Scales of Early Learning; N, number; PAS-R, Preschool Anxiety Scale-Revised; PLS-5, Preschool Language Scales, 5th Edition; PVET, Passive-Viewing Eye Tracking Task; RBS, Repetitive Behavior Scale; RRB, Restrictive Repetitive Behaviors; SD, Standard deviation; SE: Standard Error; SoL, Sleep onset Latency; SPM-P, Sensory Processing Measure-Preschool; SPS, Sensory Profile Scale; SRS, Social Responsiveness Scale; TiB, Time in Bed; TST, Total Sleep Time; VABS-II, Vineland-Adaptive Behaviour Scale-II (maladaptive behaviors); YBOCS, Yale-Brown Obsessive Compulsive Scale.
